# Supplementary material for: Cooperation Is Not Enough—Exploring Social-Ecological Micro-Foundations for Sustainable Common-Pool Resource Use
Source: PLoS One. 2016 Aug 24;11(8):e0157796. doi: 10.1371/journal.pone.0157796 (PMC4996507; doi:10.1371/journal.pone.0157796)
Supplement: S1 Appendix — ODD+D protocol, details on model process implementation, calibration and sensitivity analysis. (PDF) [file pone.0157796.s001.pdf]

S1 Appendix. Model Description. ODD+D protocol, details on model process implementation, calibration and sensitivity analysis.

The description of the agent-based model (ABM) AgentEx follows the ODD+D (Overview, Design Concepts and Details + Decision-making) protocol [1-3]).

		Guiding questions	AgentEx
**I) Overview**	I.i Purpose	I.i.a<br>What is the purpose of the study?	The purpose of the study is to advance understanding of group processes for sustainable management of a common pool resource (CPR). In particular, to develop and test explanations of cooperation and sustainable exploitation in which the explanatory power of *social skills*, *knowledge* and *confidence in knowledge* are in focus.<br>The model aims to qualitatively reproduce and explain patterns observed in behavioral CPR laboratory (lab) experiments [4,5].
		**I.ii.b For whom is the model designed?**	Researchers interested in CPR management, social-ecological systems and/or in the methodological approach of combining behavioral experiments with ABM.
	I.ii Entities, state variables and scales	I.ii.a What kinds of entities are in the model?	• Agents representing resource users (experiment participants),<br>• A collective of 4 agents (group)<br>• Resource stock (CPR) (environment)
		I.ii.b By what attributes (i.e. state variables and parameters) are these entities characterized?	Agents: confidence in knowledge, knowledge, trust, social skills, social preferences<br>Collective: group knowledge (in case there is communication)<br>Resource stock: resource stock size<br>See Table S1A for an overview.
		**I.ii.c What are the exogenous factors/drivers of the model?**	None.
		**I.ii.d If applicable, how is space included in the model?**	NA
		I.ii.e What are the temporal and spatial resolutions and extents of the model?	One time step represents one round in the behavioral experiments. Each run consists of 14 time steps aligning to the 14 rounds in the behavioral experiments.
	I.iii Process overview and scheduling	I.iii.a What entity does what, and in what order?	Each time step:<br>1. **Communicate-and-form-group-knowledge** (agent & collective)<br>2. **Update-individual-knowledge** (agent)<br>3. **Calculate-individual-extraction-level** (agent)<br>4. **Calculate-group-extraction-level** (collective)<br>5. **Choose-extraction-level** (agent)<br>6. **Extract** (agent)<br>7. **Regrow-resource-stock** (resource stock)<br>8. **Update-confidence-trust** (agent)<br>See Fig A for the process diagram and Table B for details and the formalization of these processes.
**II) Design Concepts**	**II.i Theoretical and Empirical Background**	II.i.a Which general concepts, theories or hypotheses are underlying the model's design at the system level or at the level**(s)** of the sub-model**(s) (apart from the decision model)?** What is the link to complexity and the purpose of the model?	• Social-ecological systems approach to the study of CPR systems [6].<br>• Resource dynamics: the logistic growth function for renewable resources, as often used in the resource economics literature to model resource growth (see, e.g., [7]).<br>• The model design is directly related to the setup and observations of the behavioral experiment [4, 5].
		**II.i.b On what assumptions is/are the agents' decision model(s) based?**	Assumptions based on established theory/literature:<br>• *Bounded rational* agents [8]. Being bounded rational implies that agents have limited knowledge and/or cognitive capacity to process/assess the optimal extraction level. In AgentEx, the agents have *limited knowledge* of the 'objective' optimal

			resource stock size and they do not know how the others will act beforehand.<br>• *Social preferences* and *trust* are both important for initiating and sustaining cooperation in CPR contexts (e.g. [9-11]). In AgentEx, agents can have social preferences, which implies that they are more likely to choose the group exploitation level. It also implies a preference for equal sharing of the resource (for details see E. Choose-extraction-level and C. Calculate-individual-extraction-level in Table B and Figure B).<br>Many assumptions of the agent's decision model are based on the observations of the behavioral experiments. They affected how the model was initialized and how variable relations were specified (design).<br>Initialization assumptions:<br>• Not all experiment participants understood what the optimal stock size was, i.e., the stock size that provided maximum yields. The agents therefore vary in their knowledge, i.e., what they think is optimal.<br>• One person can strongly influence group outcomes if the majority of the group has little confidence about its knowledge of the optimal stock size. This empirical observation informed the design of the scenario experiments.<br>Design assumptions on the specification of inter-individual factors relations:<br>• Speaking up - sharing knowledge - is more likely when an individual has high social skills. (paper: - Assumption 1).<br>• The higher the confidence in knowledge of an individual, the more influence the individual has on the formulation of the group agreement. (paper - Assumption 2a).<br>• The lower the confidence in knowledge of an individual, the more likely she is influenced by the opinion of others and, hence, updates her knowledge. (paper - Assumption 2b).<br>• Confidence in knowledge decreases (increases) when there is a discrepancy (match) between the actual and the expected stock size; the larger the deviation between actual and expected stock size, the stronger the decrease in confidence. (paper - Assumption 3).
		**II.i.c Why is a/are certain decision model(s) chosen?**	The decision-making process underlying the model reflects the integration of primarily observations and data from behavioral experiments complemented with relevant literature. The selection of the model elements is related to its potential to explain the patterns observed in the experiments.
		**II.i.d If the model/submodel (e.g. the decision model) is based on empirical data, where does the data come from?**	From the behavioral experimental studies of Lindahl et al. [5] and Schill et al. [4]. Data from the behavioral experiments themselves, post-experimental questionnaires and notes the experimenters took during the course of the experiments.
		**II.i.e At which level of aggregation were the data available?**	At the individual, and group and system levels.
	**II.ii Individual Decision-Making**	**II.ii.a What are the subjects and the objects of decision-making? On which level of aggregation is decision-making modeled? Are multiple levels of decision-making included?**	Agents make individual decisions on how many resource units they want to harvest (*extract*). This implies choosing between the individual and group extraction level.

		**II.ii.b What is the basic rationality behind agents' decision-making in the model?** Do agents pursue an explicit objective or have other success criteria?	The (implicit) objective of the agents is to extract resources. The decision of how much to extract depends on their knowledge, trust and social preferences.
		**II.ii.c How do agents make their decisions?**	See Fig B.
		**II.ii.d Do the agents adapt their behavior to changing endogenous and exogenous state variables? And if yes, how?**	Yes, agents change their behavior (amount of resource units taken out) based on their knowledge and trust, both can change over time.<br>See Table B for detailed description and formalization of these changes.
		**II.ii.e Do social norms or cultureal values play a role in the decision-making process?**	*Social norms* play a role in the form of social preferences and trust. Having social preferences reflects an injunctive norm [12], behavior is affected by what the agent thinks it ought to do. Trust reflects a descriptive norm, i.e. behavior is affected by the behavior that is observed, the norm of what is done.
		**II.ii.f Do spatial aspects play a role in the decision process?**	No.
		**II.ii.g Do temporal aspects play a role in the decision process?**	No.
		**II.ii.h To which extent and how is uncertainty included in the agents' decision rules?**	Both perceived environmental and social **uncertainty** play a role. Perceived environmental uncertainty is reflected by the variable confidence in knowledge. Social uncertainty is included by the fact that the agents do not know whether other agents will stick to the agreed group extraction level. Trust reflects how the agents perceive this social uncertainty.
	II.iii Learning	II.iii.a Is individual learning included in the decision process? How do individuals change their decision rules over time as consequence of their experience?	Individual learning as defined in terms of changing decision rules: No. Decision rules stay constant, just the values of the most variables change (see II.ii.d).
		**II.iii.b Is collective learning implemented in the model?**	No. However, the group adapts its group knowledge each time step based on the updated knowledge of the group members that communicate.
	II.iv **Individual** Sensing	II.iv.a What **endogenous** and **exogenous** state variables are individuals assumed to sense and consider in their decisions? **Is the sensing process erroneous?**	Resource stock: each agent senses the resource stock size (endogenous state variable) without error.
		II.iv.b What state variables of which other individuals can an individual perceive? **Is the sensing process erroneous?**	Knowledge of other agents: agents sense the knowledge of those agents that communicate. They sense the group knowledge without error.
		II.iv.c What is the spatial scale of sensing?	Global.
		II.iv.d Are the mechanisms by which agents obtain information modeled explicitly, or are individuals simply assumed to know these variables?	Agents are simply assumed to know the stock size and group knowledge. They can only know the knowledge of other agents if they communicate.
		**II.iv.e Are the costs for cognition and the costs for gathering information explicitly included in the model?**	No cost.
	II.v **Individual** Prediction	**II.v.a Which data uses the agent to predict future conditions?**	Agents do not predict future conditions.
		II.v.b What internal models are agents assumed to use to estimate future conditions or consequences of their decisions?	Agents assume that others behave as themselves when there is no communication.

		**II.v.c Might agents be erroneous in the prediction process, and how is it implemented?**	NA
	II.vi Interaction	II.vi.a Are interactions among agents and entities assumed as direct or indirect?	Direct interaction both with the resource (through extraction) and between the agents (through communication).
		**II.vi.b On what do the interactions depend?**	The direct interactions between agents depend on whether an agent communicates.
		II.vi.c If the interactions involve communication, how are such communications represented?	All agents always listen to the others. Communication involves sharing one's knowledge; the result of communication is the group knowledge.
		**II.vi.d If a coordination network exists, how does it affect the agent behavior? Is the structure of the network imposed or emergent?**	NA
	II.vii Collectives	II.vii.a Do the individuals form or belong to aggregations that affect, and are affected by, the individuals? Are these aggregations imposed by the modeler or do they emerge during the simulation?	The four agents form a group, which is imposed by the modeler. If they communicate, they form group knowledge that determines the group extraction level. If all agents follow the group extraction level they are a cooperative group.
		II.vii.b How are collectives represented?	A group is not explicitly represented. Group knowledge is a global variable.
	**II.viii Heterogeneity**	**II.viii.a Are the agents heterogeneous? If yes, which state variables and/or processes differ between the agents?**	The agents are heterogeneous in respect to the following state variables (see Table A):<br>• social preferences (true/false)<br>• social skills [0-1]<br>• trust [0-1]<br>• individual knowledge [0-50]<br>• confidence in knowledge [0-1]
		**II.viii.b Are the agents heterogeneous in their decision-making? If yes, which decision models or decision objects differ between the agents?**	The (decision) processes are the same for all agents, due to the heterogeneous attributes they just lead to different behaviors.
	II.ix Stochasticity	II.ix.a What processes (including initialization) are modeled by assuming they are random or partly random?	There are various sources of stochasticity in the model:<br>Initial settings (agent attributes):<br>• socialSkills_distribution<br>• socialPreference_distribution,<br>• init_trust_distribution,<br>• init_confidenceKnowledge_distr,<br>• init_knowledge_distr<br>During the run (processes):<br>The following processes are partly random:<br>• update-individual-knowledge<br>• calculate-individual-extraction-level<br>• choose-extraction-level<br>See Table B for details.
	II.x Observation	II.x.a What data are collected from the ABM for testing, understanding, and analyzing it, and how and when are they collected?	Mean stockSizeBeforeGrowth, Integer [0,50]: resource stock units measured before regrowth after extraction. Average stock size calculated at the end of the run.<br>coopPower, Float [0,1]: represents ratio of cooperation of a run by dividing # ticks agents cooperate (all choose group extraction level) with # ticks in total. Calculated at the end of the run.
		II.x.b What key results, outputs or characteristics of the model are emerging from the individuals? (Emergence)	**Cooperation**, per tick if the agents all choose the group extraction level AND aggregated to a run when the group extraction level is chosen for at least 70% of the time steps.<br>**Resource stock size (exploitation pattern)** as consequence of what the agents extract.

III) Details	**II.i Implementation Details**	**III.i.a How has the model been implemented?**	Netlogo 5.3.1 [13]
		**III.i.b Is the model accessible and if so where?**	Yes, it is available on openABM (www.openabm.org).
	III.ii Initialization	III.ii.a What is the initial state of the model world, i.e. at time t=0 of a simulation run?	The simulation starts at t=0: the 4 agents are initialized based on the initial distributions of social preferences, social skills, confidence in knowledge, trust and individual knowledge. The renewable CPR has an initial stock size of 50 and a set regeneration rate (see Fig in Table B).
		III.ii.b Is initialization always the same, or is it allowed to vary among simulations?	Depending on the experimental setup, the initialization varies. To build confidence in the model, we varied the initial settings broadly (extremes and middle values) to test homogenous and heterogeneous group compositions, leading to 6480 unique configurations, see Table C. To explore the impact of group compo-sition, we designed specific scenarios (focusing on configurations that were classified as cooperative) guided by the questions:<br>1) What difference can (one) informed confident agent(s) make? (Scenario set I)<br>2) What effect do opposing agents, in respect to their knowledge, have? (Scenario set II)<br>Fig C describes each of the 7 group compositions that were used.<br>Furthermore, whether an agent will speak during a simulation run is based on a social skill dependent probability, i.e., the higher the social skills the more likely the agent speaks up. This is determined once, at the beginning of the simulation run.
		III.ii.c Are the initial values chosen arbitrarily or based on data?	The choice of the initial values is driven by either the design of the experiments, observations in the behavioral experiments (see Table C). The other variables have been calibrated (see sensitivity analysis).
	III.iii Input Data	III.iii.a Does the model use input from external sources such as data files or other models to represent processes that change over time?	No.
	III.iv Submodels	III.iv.a What, in detail, are the submodels that represent the processes listed in 'Process overview and scheduling'?	See Table B.
		III.iv.b What are the model parameters, their dimensions and reference values?	Overview of the model variables, parameters, and their values are given in Table S1C.
		III.iv.c How were submodels designed or chosen, and how were they parameterized and then tested?	Table B specifies the formalization of each process. The calibration and sensitivity analysis are given below.

Table A. Overview of Main Variables and Parameters Used in AgentEx.

	**Name**	**Definition**	**Value range**
**Agent**	**Individual knowledge**	The agent's perception of the optimal resource stock size based on its individual understanding of the resource system.	5-50
	**Confidence in knowledge**	The agent's confidence in its knowledge of the resource system.	0.0-1.0
	**Trust**	The agent's belief that the other agents (group members) act as agreed.	0.0-1.0
	**Social skills**[a]	The probability that the agent speaks up, i.e., shares its knowledge with other group members (independent of the group members' actions).	0.0-1.0

	**Social preferences**[a]	The agent has a preference for equal sharing of the resource (or not).	True/False
**Group**	**Group knowledge**	The group's perception of the optimal resource stock size based on the common understanding of the resource system (reflected in the group agreement).	5-50
**Environment**	**Resource stock size**	Stock size in resource stock units.	0-50

[a] These agent attributes do not change over the course of the run.

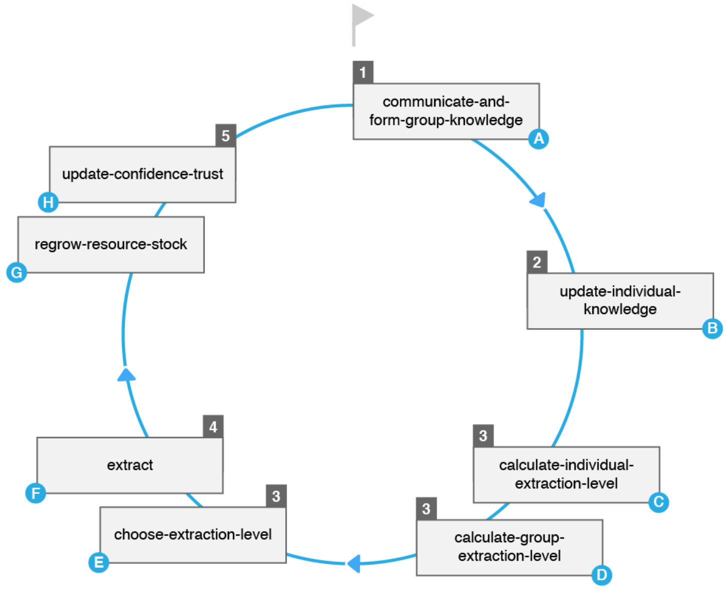

Fig A. Process Diagram AgentEx. See Table B (below) for details.

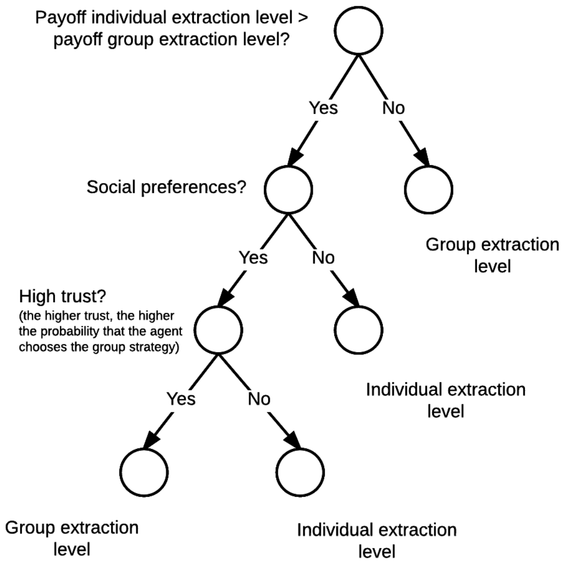

Fig B. Decision Tree of the Main Decision on How Much to Extract. Process 'choose-extraction-level'.

Table B. Details of the Implementation of the Model Processes.

Process	Variables involved	Description	Formalization
**A. Communicate-and-form-group-knowledge**	`Confidence in knowledge` *(confidence)*<br>`Individual knowledge` *(indivKnowl)*<br>`Group knowledge` *(groupKnowl)*	Not all agents speak up to share their individual knowledge with the other agents. Only those that speak take part in the formulation of the group knowledge (agreement). Whether an agent communicates depends on its social skills, inde-pendently of the actions of other group members. This is determined at the beginning of a run and is implemented as a probability, i.e., the higher the agent's social skills, the higher the likelihood that it will communicate. The knowledge of the group is calculated as a weighted average of the individual knowledge of those agents that communicate. Each agent's confidence defines the weight of its individual knowledge. This reflects the experimental observation that individuals that are very confident have more influence on the group agreement. Group knowledge reflects what the group perceives as the optimal resource stock size and determines the group exploitation level (see formulate-group-extraction-level).	`IF` (*speakers* > 0) `THEN`<br>$sumConf = \sum_{n=1}^{\#speakers} confidence_n$<br>$groupKnowledge = \frac{\sum_{n=1}^{\#speakers}(indivKnowl_i * \frac{confidence_n}{sumConf})}{\#speakers}$<br>`ELSE`<br>;no groupKnowledge<br><br>Note: speakers are agents that communicate and share their knowledge with the other agents.
**B. Update-individual-knowledge**	`Individual knowledge` *(indivKnowl)*<br>`Confidence in knowledge` *(confidence)*<br>`Group knowledge` *(groupKnowl)*	The agents update their individual knowledge mainly based on what has been said (group knowledge). The degree of change towards the group knowledge depends negatively on its confidence: the lower the agent's confidence, the stronger the update. There is always a small chance that the agent does not update towards the group knowledge, but randomly or not at all. This reflects the possibility that individuals might not be or are differently affected by others. If this is the case, the agent updates its knowledge randomly or not at all given a confidence-based probability (the higher confidence, the lower the chance of updating).<br>If there is no communication, whether the agent will update (randomly) depends on its confidence: the higher the confidence, the lower the likelihood that it randomly updates. A random update of knowledge means in the range of the initial knowledge distribution (which is discrete and uniform), e.g., [25-29] or [10-29].	`IF` (*speakers* > 0) `THEN`<br>`IF` (rnd <= 0.20) `THEN`<br>$indivKnowl = rnd(min, max)$<br>`ELSE`<br>$indivKnowl = (1 - confidence)^2 * (groupKnowl - indivKnowl) + indivKnowl$<br>`ELSE IF` (rnd > confidence) `THEN` ;no speakers<br>$indivKnowl = rnd(min, max)$<br>`ELSE` ;no change<br><br>rnd = sample from uniform(0,1) or uniform(min,max) pseudo-random number generator.
**C. Calculate-individual-extraction-level**	`Social preferences*` *(socialPref)*<br>`Planned extraction` *(planExtract)*<br>`Resource stock size` *(stock)*	The process calculate-individual-extraction level is based on the individual knowledge of the agent and its social preferences. The individual extraction level corresponds to the current stock size minus the agent's individual knowl-edge divided by the number of agents *if* the agent has social preferences. The individual extraction level of an agent without social preferences is its equal share plus a random amount of resource stock units between the	`IF` (*stock* < *indivKnowl*) `THEN`<br>*planExtract* = 0<br>`ELSE IF` (*socialPref*) `THEN`<br>$planExtract = \frac{stock - indivKnowl}{\#agents}$

	Individual knowledge<br>*(indivKnowl)*	actual resource stock size and its individual knowledge. The individual extraction level can be to extract nothing in order to allow the resource to recover to the stock size the agent perceives as optimal.	ELSE<br>$planExtract = \frac{stock - indivKnowl}{\#agents} + rand(stock, indivKnowl)$
**D. Calculate-group-extraction-level**	Group planned extraction<br>(*groupPlanExtract*)<br>Resource stock size<br>*(stock)*<br>Group knowledge<br>*(groupKnowl)*	The process calculate-group-extraction-level is based on the group knowledge. Its computation is the same as for calculate-individual-extraction-level: the group extraction level corresponds to extraction of what the group perceives to be the optimal stock size. The total resource units to be extracted are divided equally among the agents.<br>The group extraction level can be zero in order to allow the resource to recover to the stock size.	IF (stock < groupKnowl) THEN<br>*groupPlanExtract* = 0<br>ELSE<br>$groupPlanExtract = \frac{stock - groupKnowl}{\#agents}$
**E. Choose-extraction-level**	Actual extraction<br>*(actualExtract)*<br>Group planned extraction<br>*(groupPlanExtract)*<br>Planned extraction<br>*(planExtract)*	The agent chooses its extraction level (individual or group). Firstly, the choice of the agent depends on whether the individual extraction level provides a higher payoff (more resource units to extract) than the group extraction level. If the group extraction level is higher it is chosen, otherwise the individual extraction level is chosen, unless the agent has social preferences and high trust. More specifically, an agent with social preferences chooses the less profitable group extraction level based on a probability based on the agent's value of trust. An agent without social preferences chooses the individual extraction level regardless the trust level. Note that the individual extraction level differs between agents with and without social preferences. An agent with social preferences takes out its equal share, whereas an agent without takes more than its equal share.	$actualExtract = \{groupPlanExtract, planExtract\}$<br>See decision-tree in Fig B.
**F. Extract**	Resource stock size<br>*(stock)*<br>Actual extraction<br>*(actualExtract)*<br>Total extraction<br>*(totalExtract)*	Agents extract the resource units according to their decision taken in the process choose-extraction-level.	$totalExtract = \sum_{n=1}^{\#agents} actualExtract_n$<br>IF (*totalExtract* > *stock*) THEN<br>FOREACH agent<br>$actualExtract = \frac{actualExtract}{totalExtract} * stock$<br>*totalExtract = stock(t-1)*

**G. Regrow-resource-stock**	`Resource stock size` *(stock)*	The resource dynamics is represented by a discrete logistic growth function. It regenerates according to the resource dynamics illustrated in the figure to the right. The minimum resource stock size necessary for regeneration is five units. The maximum resource stock size is 50 units (carrying capacity). The regeneration rate changes in steps of five units and the maximum sustainable yield is nine resource stock units.	stockBeforeGrowth[R] = round(stock – totalExtract)<br>growth = lookup (resource dynamics, stockBeforeGrowth)<br>stock = stockBeforeGrowth + growth<br>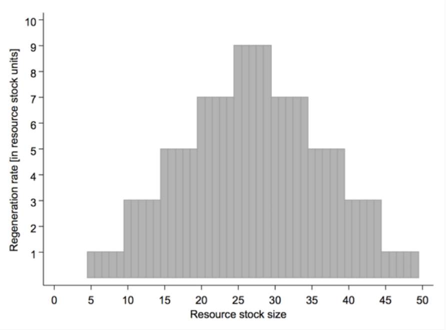
**H. Update-confidence-trust**	`Confidence in knowledge` *(confidence)*<br>`Trust`<br>`confTust_updateConst`[c]<br>`deviationSensitivity`[c]	The agent uses information it receives about the new state of the resource to update its confidence in knowledge and trust in other agents' to stick to the group agreement. Each agent has an expectation of the stock size based on its own extraction and the expected extraction of the other agents. If the stock size corresponds to what the agent expects, its trust and confidence increases. If the new stock size is larger than expected, confidence decreases, as the agent attributes this deviation to lack of knowledge. Trust does not change in that case, taking less than agreed is not considered to harm trust. If stock size is smaller than expected both trust and confidence decrease, as the agent cannot be sure whether the deviation is due to because someone has taken more (other) or because of lack of knowledge (self). The magnitude of the negative correction of trust and confidence depends on the size of the deviation, i.e., the bigger the deviation, the stronger the decrease of trust and confidence.	`IF` (speakers > 0) `THEN`<br>expectedExtraction = (#agents-1) * groupExtraction + my-actualExtraction<br>`ELSE` ;no communication<br>expectedExtraction = #agents * plannedExtraction<br>expectedStock = stock(t-1) – expectedExtraction<br>expectedGrowth = lookup (resource dynamics, expectedStock)<br>expectedStock = expectedStock + expectedGrowth<br>deviation[R] = round(stock – expectedStock)<br>change = confTrust_updateConst * deviation * deviationSensitivity<br>`IF` (deviation < 0) `THEN`<br>confidence = confidence - change<br>trust = trust – change<br>`ELSE IF` (deviation > 0) `THEN`<br>confidence = confidence - change<br>`ELSE` ;no deviation<br>trust = trust + change<br>confidence = confidence + change

[c] Constants, see Calibration section (below) for details.
[R] Variable is rounded off to the nearest integer.

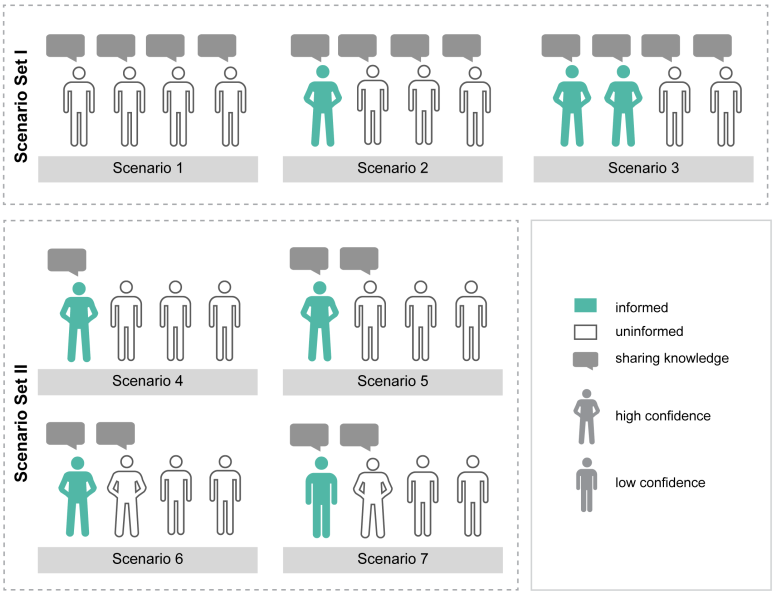

Fig D. Visual Description of Developed Scenarios in Two Sets to Explore the Impact of Group Composition. (Not) sharing knowledge implies that social skills are set to a value 1 (0). Informed agents are initialized with an individual knowledge value between [25-29] and uninformed agents between [5-24] respectively. High (low) confidence implies an initial value of 0.8 (0.2). Non-speakers always have low confidence and are uninformed to ensure tractability. Agents that do not speak up and share their knowledge will not influence the group knowledge. In all scenarios, all agents have social preferences and are initialized with high trust values [0.66-0.94]. See Table 1 and Table C (below) for details.

Table C. Initial Settings of Agent Attributes Used for the Simulation Experiment to Gain Confidence in the Model and Motivation for Chosen Initial Settings. Please note that the initial settings represent group compositions in respect to the individual attribute. A low value can be any value between [0.06-0.34]; medium value can be any value between [0.36-0.64]; high value can be any value between [0.66-0.94].

Name	Type	Value range	Initial settings	Motivation for initial settings
Social skills[a]	Float	0-1	1) all agents have high value<br>2) all agents have low value<br>3) all agents have value 0<br>4) 75% of agents have low value, 25% have high value<br>5) 50% of agents have low value, 50% have high value<br>6) 25% of agents have low value, 75% have high	The experimenters observed (not measured) that groups varied quite a bit in respect to how many subjects spoke up and shared their knowledge with the others. In order to explore the effect of communication, we decided to vary the initial settings of social skills so to have homogenous and heterogeneous groups. We chose six different initial settings: homogenous groups in which 1) all agents have high, 2) all agents have low and 3) none have social skills; as well as heterogeneous groups in which the 4) majority has high and minority has low social skills and 5) vice versa as well as 6) half has high and half has low social skills.
Social preferences[a]	Boolean	True/ false	1) all true<br>2) 75% true<br>3) 50% true<br>4) 25% true<br>5) none true	Lindahl et al. [5] and Schill et al. [4] observed a strong equal sharing norm in the behavioural experiments and the data from the post-experimental questionnaire indicated that for the majority of the subjects fairness played a role in their decision-making. Subjects were asked to indicate on a Likert-scale from one to five (where five means strongly agree) to what extent they agree with the following statement: "Fairness played a role in my decision-making." To be able to explore the role of social preferences in configurations in which all agents have optimal knowledge from the beginning, we also chose initial distributions in which only half, the minority or no agents have social preferences. We choose the following initial value settings: 1) all agents have social preferences; 2) 75% of the agents (i.e., 3 agents) have social preferences; 3) 50% (i.e., 2 agents) have social

				preferences; 4) 25% of the agents have social preferences and 5) none have social preferences.
Trust	Float	0-1	1) all agents have high value<br>2) all agents have medium value<br>3) all agents have low value<br>4) 75% of agents have low value, 25% have high value<br>5) 50% of agents have low value, 50% have high value<br>6) 25% of agents have low value, 75% have high	The sensitivity analysis for the initial trust settings showed strong influence. Moreover, data from the post-experimental questionnaire "Generally speaking, I only trust people that I have known for a while." indicates that there is quite a variety across the subjects in respect to this statement. Therefore, we test 16 different initial trust settings from random to homogenous high, medium and high trust settings to heterogeneous groups.
Confidence in knowledge	Float	0-1	1) all agents have high value<br>2) all agents have medium value<br>3) all agents have low value<br>4) 75% of agents have low value, 25% have high value<br>5) 50% of agents have low value, 50% have high value<br>6) 25% of agents have low value, 75% have high	The experimenters observed (not measured) that the subjects varied substantially in their confidence in respect to their knowledge. Therefore, we seek to vary it equally broad as the initial trust, see above.
Initial knowledge	Integer (drawn from a uniform distributio n)	5-50	1) 25-29 (perfect knowledge)<br>2) 20-34 (good knowledge)<br>3) 15-39 (skewed over-exploitation knowledge)<br>4) 25-44 (skewed under-exploitation knowledge)<br>5) 10-29 (larger range)<br>6) 5-49 (complete range)	We chose these settings to be able to reflect (1) relatively homogenous groups in which all agents know what is optimal; (2-3) more heterogeneous groups that can include agents with optimal as well as more (3) and less (2) over- and under-exploitation; (4) heterogeneous groups with a tendency for under-exploitation or (5) overexploitation; and (6) large heterogeneous groups ("anything goes").
**Constants**[*]				
confTrust_ update_ constant	Float		0.07	A value of 0.07 implies that an agent starting with no confidence or trust would get to a value of almost 1 (0.98) by the end of the game if there was no deviation throughout the game.
deviation Sensitivity	Float		0.333	This value implies that when the absolute deviation (between what the agent expects to be the new resource stock size and the actual resource stock size) is three, the agent updates its trust (only of there is a negative deviation) and confidence to the same extent (just in a negative direction) as if there was no deviation, i.e., according to the value of the confTrust_update_constant. A sensitivity analysis with values of 0.5 and 1 showed little change, see Calibration and Sensitivity Analysis section below.
probIND	Float		0.8	If there is communication, by a probability of 0.8 the agent updates its knowledge (change depends on confidence) toward the group knowledge.
[a]Agent attributes that do not change over time.<br>[*]See calibration and sensitivity analysis section below.				

Calibration

probIND, confTrust_update_constant and deviationSentivity are constants. Below we shortly explain how we calibrated each of them.

probIND is part of the 'update-individual-knowledge' function. It makes sure that there is always a small chance that an agent changes its knowledge randomly and not towards the group knowledge. probIND is set to 0.8, implying that 20% of the time the update will not change towards group knowledge. Results of a sensitivity analysis did not show a meaningful overall effect of this parameter.

confTrust_update_constant indicates the magnitude of change when changing the confidence and trust in the ‘update-confidence-trust’ function. It is set to 0.07 to allow trust/confidence to increase to (almost) 1 during one simulation run (14 time steps) if trust/confidence is initially 0 or close to zero.

deviationSensitivity represents the strength of influence on the increase or decrease of trust or confidence when the resource stock size deviates from what the agent expected it to be. deviationSensitivity is part of the ‘update-confidence-trust’ function: {trust, confidence} - confTrust_update_constant * deviation * deviationSensitivity
It is set to 0.333 although it does not make much difference, see Figure S1D below.

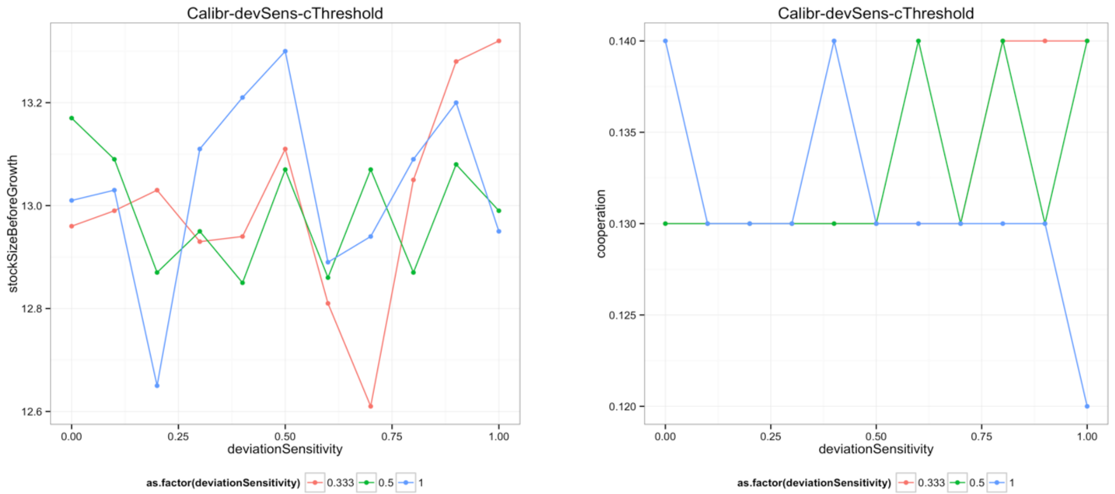

Fig D. Calibration of Variables confTrust_update_constant and deviationSensity

Reasoning: A value of 0.333 (and conTrust_update_constant = 0.07) leads to the following: In case of a deviation of 10 resource stock size units (which changes the regeneration rate of the resource by four units), leads to a change in trust/confidence of 0.23 and a deviation of 15 to 0.35. We think trust and confidence should erode much more with having such a large deviation. In future steps we will explore other functions than linear change.

Sensitivity analysis: Number of runs (repetitions)

To calculate the number of runs we used standard error of means (SEM), following Ritter et al. [14], see Eq. 1 and visual inspection of the cumulative mean of the outcome variables.

$$SEM = Variance / N = Standard\ deviation / sqrt(N) \quad (Eq.\ 1)$$

We determined that the number of runs for this experiment should be minimally 1000 repetitions, see Figure S1E. This is based on a confidence interval of 5% and a sensitivity for the response variables that allows distinguishing between a stock size +/- 1 and cooperation probability +/- 1%. The simulation experiments and results are based on 5000 repetitions.

The figures below provide an impression about the stabilization over runs, and restates - the more runs the better [14].

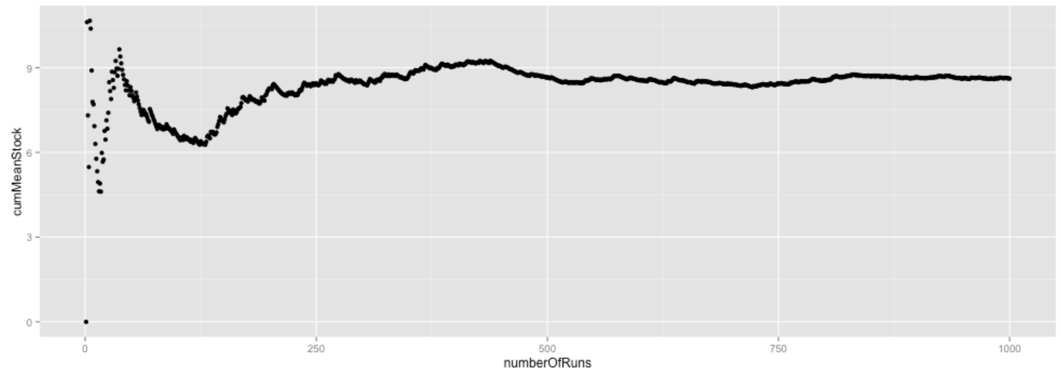

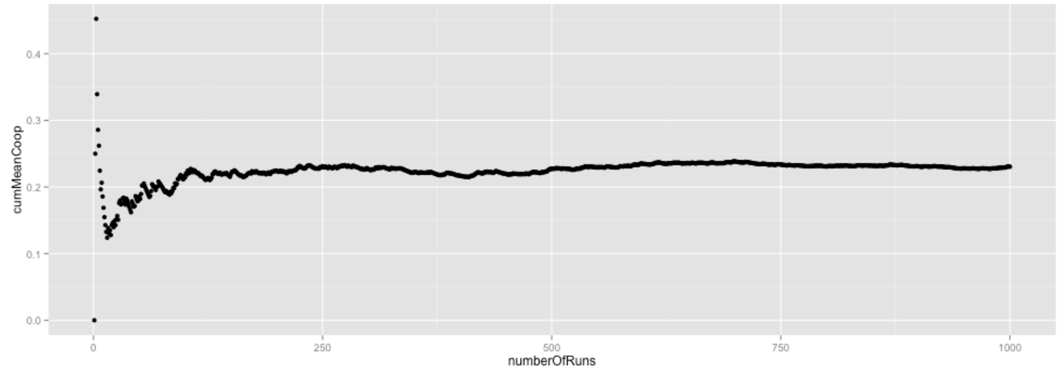

Fig E. Stabilizing Cumulative Mean Stock Size and Cooperation while increasing the Number of Runs.

References

1. Müller B, Bohn F, Dreßler G, Groeneveld J, Klassert C, Martin R, et al. Describing human decisions in agent-based models - ODD + D, an extension of the ODD protocol. Environmental Modelling & Software. 2013;48(C):37–48.
2. Grimm V, Berger U, Bastiansen F, Ostrom E, Ginot V, Giske J, et al. A standard protocol for describing individual-based and agent-based models. Ecological Modelling. 2006;198(1-2):115–26.
3. Grimm V, Berger U, DeAngelis DL, Polhill JG, Giske J, Railsback SF. The ODD protocol: A review and first update. Ecological Modelling. 2010;221(23):2760–8.
4. Schill C, Lindahl T, Crépin A-S. Collective Action and the Risk of Ecosystem Regime Shifts: Insights from a Laboratory Experiment. Ecology and Society. 2015;20(1).
5. Lindahl T, Crépin Anne-Sophie, Schill C. Potential disasters can turn the tragedy into success. Environmental and Resource Economics. DOI 10.1007/s10640-016-0043-1.
6. Berkes F, Carl Folke, editors. Linking Social and Ecological Systems. Cambridge, UK: Cambridge University Press; 1998.
7. Clark CW. Mathematical bioeconomics: the optimal management of renewable resources. Second. New York: John Wiley & Sons, Inc; 1990.
8. Simon HA. Models of Man, Social and Rational: Mathematical Essays on Rational Human Behavior in a Social Setting. John Wiley and Sons, Inc; 1957.
9. Ostrom E. A behavioral approach to the rational choice theory of collective action: Presidential address, American Political Science Association, 1997. American Political Science Review. 1998;92:1–22.
10. Fehr E, Schmidt KM. A theory of fairness, competition, and cooperation. The quarterly journal of economics. 1999;114(3):817–68.
11. Fehr E. On The Economics and Biology of Trust. Journal of the European Economic Association. 2009;7(2-3):235–66.
12. Cialdini RB, Reno RR, Kallgren CA. A Focus Theory of Normative Conduct: Recycling the Concept of Norms to Reduce Littering in Public Places. Journal of Personality and Social Psychology. 1990;58(6):1015–26.
13. Wilensky U. Netlogo. Evanston, IL: Center for Connected Learning and Computer-Based Modeling Northwestern University.
14. Ritter FE, Schoelles MJ, Quigley KS, Klein LC. Determining the number of simulation runs: Treating simulations as theories by not sampling their behavior. Springer; 2011;97–116.
