# Supplementary material for: Cooperation Is Not Enough—Exploring Social-Ecological Micro-Foundations for Sustainable Common-Pool Resource Use
Source: PLoS One. 2016 Aug 24;11(8):e0157796. doi: 10.1371/journal.pone.0157796 (PMC4996507; doi:10.1371/journal.pone.0157796)
Supplement: S2 Appendix — (PDF) [file pone.0157796.s002.pdf]

S2 Appendix. Supplementary Information on Simulation Experiments. Screenshot NetLogo model, details on building confidence in model simulation experiment and information on pattern classifications of simulation experiment outcomes.

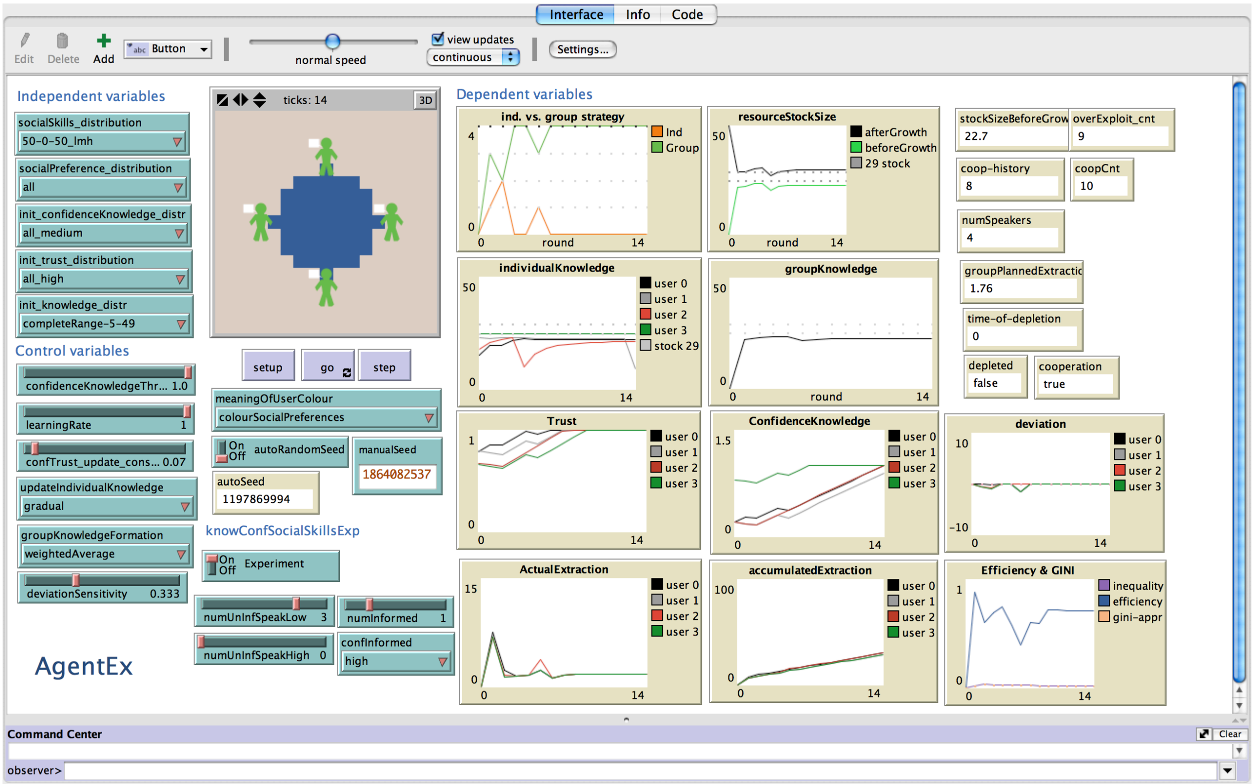

Fig F. Screenshot Scenario 2 (Scenario Set I) of NetLogo AgentEx Model. See Fig 5 for initial settings and values used specifically for this scenario and Table 1 (paper) and Table C in S1 Appendix for definitions and value ranges of all other model components. Note, comparing the *group knowledge* graph with the *individual knowledge* graph indicates that in some time steps the *individual knowledge* of an uninformed agent, e.g., user 2, is lower than the *group knowledge*. This means, that in those time steps, the agents would have a higher payoff by choosing their individual extraction level. However, since all agents have *social preferences* and the *trust* of all agents is relatively high from the beginning, the agents chose in all but three time steps the group extraction level, which classifies this run as cooperative. This is shown in the upper left graph which provides information on how many agents choose the individual versus the group extraction level. *Trust* and *confidence in knowledge* increase as agents cooperate. *Trust* of all agents reaches the maximum value of one after about half of the time steps, which results in cooperation for the rest of the time steps. Increasing *confidence* of the uninformed agents leads to an increasing influence of the uninformed agents on *group knowledge*, relative to the informed agent, over time. Recall, *group knowledge* is based on a weighted average of the *individual knowledge* of communicating agents (i.e., have *social skills*) depending on their *confidence*. Since they all share their respective *individual knowledge*, the *group knowledge* will be slightly lower than the perfect *individual knowledge* of the informed agent. However, due to the low *confidence* of the uninformed agents in the beginning of the run, the informed agent has most influence on the *group knowledge*, as its *confidence* is higher throughout the run (apart from the last time step). The *individual knowledge* graph also shows that in the first time steps, the *individual knowledge* of the uninformed agents approximates the *individual knowledge* of the informed agent, this relatively strong assimilation is due to the low *confidence* of the uninformed agents.

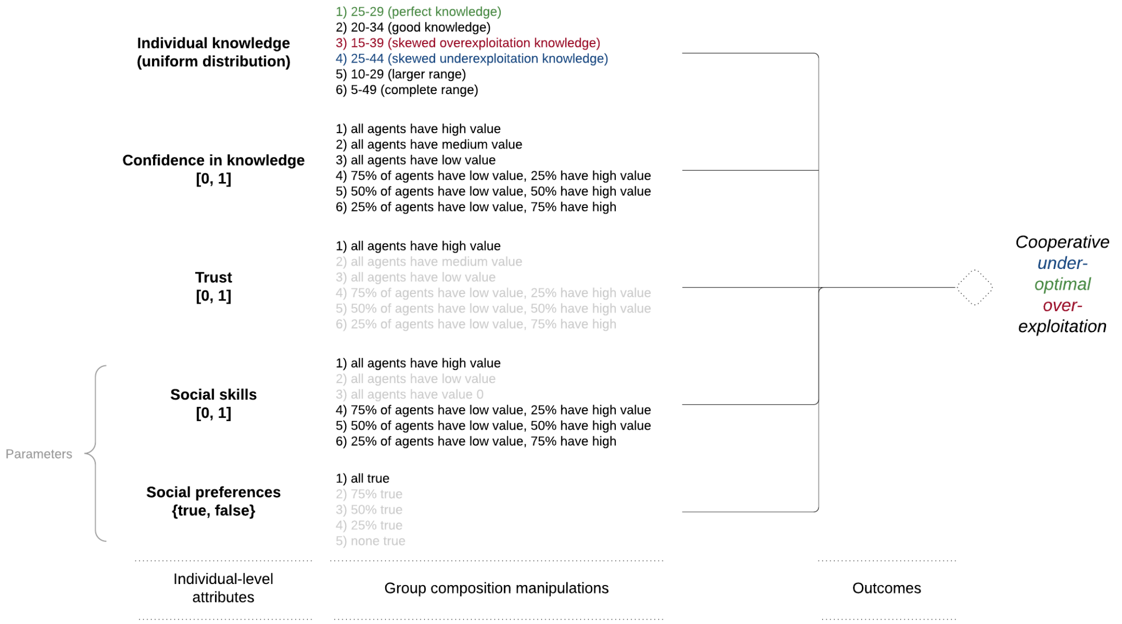

Fig G. Building Confidence in AgentEx Simulation Experiment, Initial Settings and Outcome Patterns. Initial settings (values) in relation to outcome patterns. *Social skills* are, like *trust* and *social preferences*, an important variable for cooperation. All four agents need to have a value of *social skills* > 0 for cooperation to emerge. More specifically, minimally one agent needs to have high *social skills*, i.e., a high probability of speaking up. This is due to the fact that cooperation cannot emerge without communication. In respect to *confidence in knowledge* and *individual knowledge*, we found all initial settings among the configurations that classify as cooperative. Whether or not such a configuration leads to an optimal-, over- or underexploitation outcome pattern depends only on the initial *individual knowledge* distribution. (Under-) overexploitation *knowledge* leads to under- overexploitation; perfect *knowledge* leads to optimal exploitation. *Confidence in knowledge* and *social skills* did not influence this classification. See Table 1 for definitions and value ranges of variables and parameters and Table D (below) for definitions of classifications/outcome patterns.

Table D. Overview of Patterns and Definitions of Classifications Used to Interpret the Simulation Experiment Results.

Pattern Classification	Definition
**Cooperation**	For each run, coopPower is measured, representing the ratio of cooperation by dividing # ticks agents cooperate (all choose group extraction level) with # ticks in total. If the average coopPower of a configuration (consists of 5000 runs each) >= 0.7, the configuration is classified as cooperative.[a]
**Non-cooperation**	If the average (coopPower-1) of a configuration >= 0.7, the configuration is classified as non-cooperative.
**Optimal exploitation**	For each run, optimal_cnt is measured, which counts # ticks after tick 5 in which CPR is optimally exploited, i.e., resource stock size between and including 25 to 29. If average optimal_cnt >= 8, the configuration is classified as optimal.
**Overexploitation**	For each run, overExploit_cnt is measured, which counts # ticks after tick 5 in which CPR is underexploited. If average overExploit_cnt >= 8, the configuration is classified as overexploiting.
**Underexploitation**	For each run, underExploit_cnt is measured, which counts # ticks after tick 5 in which CPR is underexploited. If average underExploit_cnt >= 8, the configuration is classified as underexploiting.
**Mixed exploitation strategies**	Configuration neither classified as optimal, over- or underexploitation.

[a] The threshold level of average coopPower is set to 0.7 to account for the stochasticity in the model, more specifically in the processes ‘update-individual-knowledge’, ‘calculate-individual-extraction-level‘ and ‘choose-extraction-level’, which all influence (in)directly whether or not an agent chooses the group extraction level in any given time step.
