# Supplementary material for: Cooperation Is Not Enough—Exploring Social-Ecological Micro-Foundations for Sustainable Common-Pool Resource Use
Source: PLoS One. 2016 Aug 24;11(8):e0157796. doi: 10.1371/journal.pone.0157796 (PMC4996507; doi:10.1371/journal.pone.0157796)
Supplement: S3 Appendix — (PDF) [file pone.0157796.s003.pdf]

S3 Appendix. Supplementary Information on Behavioral Experiments Data.

Common-pool resource game data

The exploitation and cooperation patterns of the behavioral experiments we used for this study are the observations of the ‘no threshold’ treatment from the study by Lindahl et al. [1], see Fig 1 (main text) for the resource dynamics of this treatment. We choose this treatment, as we wanted to keep the resource dynamics of the ABM *fairly* simple for tractability reasons. Lindahl et al. report about 21 ‘no threshold’ groups. For the purpose of this study, we excluded groups that depleted the resource in agreement (two groups) in order to keep our explanation (i.e., model) simpler.

Post-experimental questionnaire and experimenter notes data

To inform the agent-based model (ABM) and the initial settings and values for the simulation experiment to build confidence in the model, we complemented the data of the above mentioned observations with data from post-experimental quesitonnaires and experimenter notes from the studies by Lindahl, Schill and collegues [1, 2]. We used the following statements and questions from the post-experimental questionnaires:

“Generally speaking, I express my opinion and thoughts openly and feel comfortable in discussions.” (*social skills*)

“Fairness played a role in my decision-making.” (*social preferences*)

“Generally speaking, I only trust people that I have known for a while.” (*trust*)

For these three statements, experiment participants indicated on a five-level Likert scale to what extent they (dis)agree.

Individual knowledge (optimal exploitation level): “Assume you were the only resource user, i.e., no one but you harvests the resource. What would be your harvest claim in the first round (in resource units)?”

On average, participants answered that they would harvest 21 resource units in the first round (SD=9). Please note, that this question was asked at the very end of the experiment, hence, one can assume that learning took place.

References

1. Lindahl T, Crépin A-S, Schill C. Potential disasters can turn the tragedy into success. Environ Resour Econ. DOI 10.1007/s10640-016-0043-1.
2. Schill C, Lindahl T, Crépin A-S. Collective action and the risk of ecosystemregime shifts: insights from a laboratory experiment. Ecol Soc. 2015;20(1):48.
