# Supplementary material for: Correction: Cooperation Is Not Enough Exploring Social-Ecological Micro-Foundations for Sustainable Common-Pool Resource Use
Source: PLoS One. 2016 Nov 15;11(11):e0165009. doi: 10.1371/journal.pone.0165009 (PMC5112906; doi:10.1371/journal.pone.0165009)
Supplement: S2 File — (PDF) [file pone.0165009.s002.PDF]

PLOS | ONE

RESEARCH ARTICLE

Cooperation Is Not Enough—Exploring Social-Ecological Micro-Foundations for Sustainable Common-Pool Resource Use

Caroline Schill[1,2]*, Nanda Wijermans[2], Maja Schlüter[2], Therese Lindahl[1,2]

1 The Beijer Institute of Ecological Economics, The Royal Swedish Academy of Sciences, Stockholm, Sweden, **2** Stockholm Resilience Centre, Stockholm University, Stockholm, Sweden

* caroline.schill@beijer.kva.se

CrossMark
click for updates

OPEN ACCESS

Citation: Schill C, Wijermans N, Schlüter M, Lindahl T (2016) Cooperation Is Not Enough—Exploring Social-Ecological Micro-Foundations for Sustainable Common-Pool Resource Use. PLoS ONE 11(8): e0157796. doi:10.1371/journal.pone.0157796

Editor: Angel Sánchez, Universidad Carlos III de Madrid, SPAIN

Received: January 19, 2016

Accepted: June 3, 2016

Published: August 24, 2016

Copyright: © 2016 Schill et al. This is an open access article distributed under the terms of the Creative Commons Attribution License, which permits unrestricted use, distribution, and reproduction in any medium, provided the original author and source are credited.

Data Availability Statement: All relevant data are within the paper and its Supporting Information files.

Funding: The research has received funding from the European Research Council (https://erc.europa.eu) under the European Union's Seventh Framework Programme (FP/2007-2013)/ERC grant agreement no. 283950 SES-LINK (NW, MS, www.seslink.org), the Swedish Research Council Formas (http://www.formas.se/en/) SUPER-project (#250-2010-145) (CS), Riksbankens Jubileumsfond (http://www.rj.se/en) (P12-0894:1) (TL) and a core grant to the Stockholm Resilience Centre by the Swedish Foundation for Strategic Environmental Research

Abstract

Cooperation amongst resource users holds the key to overcoming the social dilemma that characterizes community-based common-pool resource management. But is cooperation alone enough to achieve sustainable resource use? The short answer is no. Developing management strategies in a complex social-ecological environment also requires ecological knowledge and approaches to deal with perceived environmental uncertainty. Recent behavioral experimental research indicates variation in the degree to which a group of users can identify a sustainable exploitation level. In this paper, we identify social-ecological micro-foundations that facilitate cooperative sustainable common-pool resource use. We do so by using an agent-based model (ABM) that is informed by behavioral common-pool resource experiments. In these experiments, groups that cooperate do not necessarily manage the resource sustainably, but also over- or underexploit. By reproducing the patterns of the behavioral experiments in a qualitative way, the ABM represents a social-ecological explanation for the experimental observations. We find that the ecological knowledge of each group member cannot sufficiently explain the relationship between cooperation and sustainable resource use. Instead, the development of a sustainable exploitation level depends on the distribution of ecological knowledge among the group members, their influence on each other's knowledge, and the environmental uncertainty the individuals perceive. The study provides insights about critical social-ecological micro-foundations underpinning collective action and sustainable resource management. These insights may inform policy-making, but also point to future research needs regarding the mechanisms of social learning, the development of shared management strategies and the interplay of social and ecological uncertainty.

Introduction

Many livelihoods depend on the extraction of natural resources, like fish, timber or clean water, provided by local ecosystems. The tension between individual and collective interests

(http://www.mistra.org/en/) (MS). The funders had no role in study design, data collection and analysis, decision to publish, or preparation of the manuscript.

Competing Interests: The authors have declared that no competing interests exist.

that is often present among a community of natural resource users can impede cooperation, which is necessary for the long-term provision of the resources the community depends upon [1,2]. This tension arises because once a resource unit is extracted, it is no longer available to another user; and it is difficult to exclude others from the use of the same resource. These properties characterize common-pool resource (CPR) management settings (commons dilemmas), which have been the subject of study by many scholars from various fields and disciplines using an array of methodological approaches [3–9]. This has led to substantial advancements in our understanding about the conditions facilitating cooperation around the use of CPRs, such as trust and communication [10].

But does cooperation necessarily lead to sustainable CPR use? The short answer to this question is no. It does not 'only' take cooperation, but also extraction levels that correspond with the dynamics of the ecosystem providing the resource. Cooperation in a self-governed commons dilemma implies that resource users restrain their exploitation to a mutually agreed level. Given ecological complexity and associated uncertainties, identifying sustainable extraction levels is difficult and has been the focus of much research in various fields, such as fisheries science (e.g., [11]). Within the field of natural resource management, researchers have highlighted the importance of local ecological knowledge in dealing with ecosystem complexity [3,12]. However, knowledge of ecosystem dynamics and how it interacts with other individual-level factors, has received relatively little attention in the behavioral CPR literature. Recent observations from behavioral experiments confirm that cooperation itself is not enough to achieve sustainable resource use. In a series of laboratory CPR experiments the first and last author of this paper [13,14], investigated collective behavior and exploitation strategies in the presence of ecological complexity. Overall, and not surprisingly, cooperation had a positive effect on sustainable resource use. However, some cooperative groups over- or underexploited the CPR during the experiment. This suggests that cooperation may be a necessary, but not a sufficient condition for sustainable resource use. We believe that this observation deserves further attention.

The aim of this paper is to identify conditions under which cooperation amongst resource users can lead to sustainable resource use in a self-governed CPR setting. More specifically, we develop a possible explanation for cooperative sustainable resource use based on insights from behavioral experiments and relevant literature and test it against behavioral experimental patterns using an agent-based model (ABM). Once we establish confidence in our model, we perform simulation experiments in which we manipulate the *distribution* of initial values of key individual-level attributes (see below) in cooperative groups to systematically explore the implications of those manipulations for group level outcomes (i.e., exploitation patterns).

We regard a commons dilemma as a social-ecological system (SES) [12]: Resource users interact with and affect, not only each other, but also the ecosystem, on which they depend. It is these social and social-ecological interactions that determine not only the capacity of resource user groups to cooperate but also the prospects for sustainable resource use. Much work in the CPR literature focuses on social interactions and is devoted to the question of what makes cooperation emerge and persist. For example, trust and communication have received a lot of attention in the CPR literature and have been highlighted as crucial cooperation facilitators [10,15–18]. Group discussions can give a sense of group identity and build trust [19] and can reveal individuals' intentions to cooperate (or not) [20]. These mechanisms facilitate cooperation by reducing *social uncertainty*, i.e., uncertainty regarding whether others are going to act in the interest of the group. Resource users, however, also face *environmental uncertainty* resulting from a lack of knowledge about ecological processes and the state of the ecosystem at a given point in time.

Less attention in the CPR literature has been directed towards the interactions between the user group and the ecosystem (social-ecological interactions) and the effects of environmental uncertainty. User groups must have the relevant ecological knowledge in order to manage the shared resources sustainably, including an understanding or awareness of ecological complexities and uncertainties. "Ecosystems are moving targets", characterized by non-linearity, unpredictable fluctuations and uncertain futures [21]. Moreover, user groups also need to collectively act on their ecological knowledge. Sustainable ecosystem management, thus, depends on both types of interactions: social-social and social-ecological; and is influenced by social *and* environmental uncertainties.

The behavioral CPR experiments [13,14], this work is built on, highlight the importance of the individuals' understanding of the ecological system (in particular the resource dynamics and the sustainable extraction level), and whether this understanding is shared among and acted upon by the individuals in the group. Moreover, the experiments also highlight that individuals differ in their confidence of this understanding, which can be interpreted as environmental uncertainty. For the purpose of this study, we build on these insights both from the aforementioned behavioral experiments and relevant literature and identify *individual ecological knowledge* of the users in the group, their *confidence in that knowledge* and their respective *social skills* (the propensity of sharing knowledge) to be key influencing individual-level attributes for a cooperative group to manage the resource sustainably. We assume that the individuals composing a user group are heterogeneous in respect to these three attributes and argue that an *explanation* for the exploitation patterns found in the experiments lies in the *distribution* of these three key attributes within the cooperative group. In other words, group composition with respect to these attributes matters.

Developing and testing this explanation and exploring its consequences requires investigating individual attributes and individuals' personal perceptions and across different group settings–something that is difficult with the behavioral experimental method. Agent-based modeling allows us to address the *how* of the behavioral patterns we want to explore, i.e., develop a mechanism-based explanation [22]. The ABM is a formalization of our hypothesis about micro-level mechanisms that generate the phenomenon observed in the behavioral experiments in a simulation model. The model, called 'AgentEx' (***agent***-based modeling meets behavioral ***ex***periments), can then be used to simulate the behavior of the system and to systematically explore the consequences of different assumptions about key factors and interactions [22,23]. In simulation experiments we can control, measure and manipulate processes and perceptions internal to an individual, i.e., agent, and observe their effects on overall outcomes. The model allows us to relate micro-level processes to group level patterns of interest and vice versa, to investigate how group level patterns affect the behavior of the individuals [24]. This is particularly interesting in our case, where successful management is most likely determined by individual attributes (e.g., knowledge, confidence in knowledge, social skills) and group level (e.g., group composition) factors. By comparing model outcomes with empirical patterns, we test the validity of our explanation and identify those settings that lead to cooperative, sustainable resource use. In sum, these simulation experiments allow us to manipulate group composition with respect to the three individual-level attributes and thereby address conditions under which cooperation leads to sustainable resource use.

The use of ABMs to study the emergence of cooperation and sustainable resource use is not new. It has been shown to be valuable in numerous studies (see, e.g., [20] for an overview of ABMs of collective action or [25] for an overview of ABM applied to environmental management). However, most studies focus on the evolution of norms and rules, as well as the effect of different human behavior models on cooperative outcomes, rather than social-ecological interactions (notable exceptions are [26–28]). ABMs and behavioral CPR experiments have been

combined before [29–31]. These ABMs are, like ours, empirically grounded or informed by experimental data. However, none of these studies consider factors and processes concerned with communication and/or knowledge sharing between the agents. The authors of [31], for example, considered for their modeling exercise only the first round of the CPR experiments, in which communication was not allowed and individuals came together for the first time to manage a CPR. They "did this to avoid the complications of model learning and communication" (see paragraph 3.2 in [31]) Communication was allowed in the behavioral experiments our ABM is built on, and played a crucial role for cooperation. Therefore, we include processes of communication such as knowledge sharing. Furthermore, we also consider that agents may update their knowledge over time. Hence, we present here, to the best of our knowledge, the first ABM informed by empirical data of a CPR experiment that includes processes of communication, knowledge sharing and updating.

In the remainder of the paper, we will first introduce the empirical background to this study. Next, we will define and provide justifications for the following three individual-level attributes that build the core of our explanation: individual ecological knowledge; confidence in knowledge and social skills as well as the process of group knowledge formation. We then describe our model and explain the design of the simulation experiment for i) building confidence in the model and ii) exploring the consequences of our explanation for group-level outcomes (i.e., exploitation patterns) by varying group composition with respect to the three explanatory variables. After presenting the results, we will conclude the paper with a discussion and conclusions.

Empirical Background: Behavioral CPR Experiments

The empirical patterns we aim to explain are derived from behavioral dynamic laboratory CPR experiments, henceforth called 'behavioral experiments'. These behavioral experiments were designed to study collective behavior in the face of different ecological dynamics [13,14]. Groups of four individuals collectively managed a renewable resource stock. The experiments consisted of 14 rounds, which was unknown to the participants, and face-to-face communication was allowed at any stage. In each round, each participant decided anonymously and individually on the amount of resource units she would like to harvest. Where the group made use of the opportunity to communicate, the participants typically exchanged their knowledge about the resource stock size they perceived to be optimal and they jointly formulated, based on that shared knowledge, a group agreement on how many resource units each individual should take out. After each round, the experimenter calculated the new resource stock size and provided it to the group.

The experimental observations we used for this study were the 19 groups that were confronted with the resource dynamics shown in Fig 1. The resource dynamics represents a discrete version of a logistic growth function and has a maximum resource stock size of 50 units and a minimum resource stock size of 5 units to allow for regeneration. The regeneration rate changes in steps of five units, and the maximum sustainable yield (MSY) is 9 units. Each group started with a resource stock size of 50 units. The optimal exploitation strategy was to harvest 25 units together in the first round and thereafter 9 units per round (corresponding to the MSY) for as long as each group member expected the game to continue for at least one more round. If the group extraction lead to a stock size below 25 units, the optimal strategy for eventually obtaining large harvests again was to allow the resource to recover until it reached 34 units and then to extract 9 units in subsequent rounds. The experimenters made sure that everyone understood the resource dynamics (i.e., the resource regenerates depending on the stock size, which is in turn determined by the group's total extraction in the previous round).

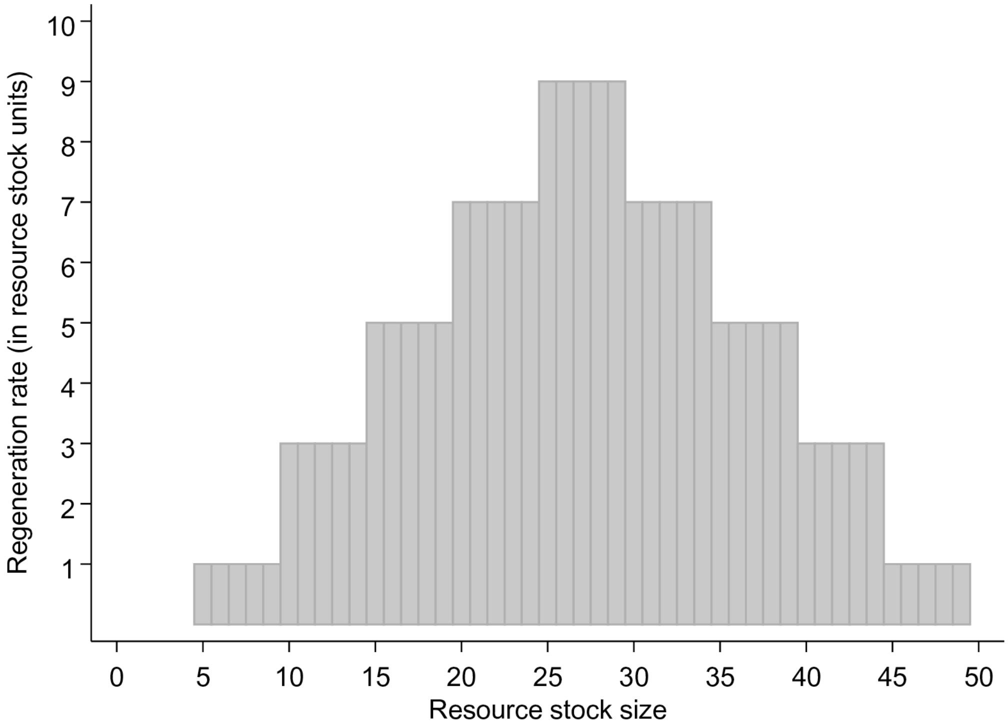

Fig 1. Graphical Illustration of the Resource Dynamics Used in the Behavioral Experiments. This graph (adapted from [14]) was presented to the experiment participants. It represents a discrete version of the logistic growth function. The minimum resource stock size that allows for regeneration is 5 units and the maximum resource stock size is 50 units. The maximum sustainable yield is nine units and the regeneration rate (in resource stock units) changes in steps of five units.

doi:10.1371/journal.pone.0157796.g001

The post-experimental questionnaires indicated that all participants did understand the resource dynamics, but that not all were able to figure out the optimal extraction level.

For the purpose of this paper, optimal exploitation is assumed to lead to sustainable resource use. We are aware that in reality, there is considerable uncertainty about actual stock size and population dynamics that makes the identification of an optimal extraction level difficult. Instead, a more cautious approach to ecosystem management is necessary [32]. In the experimental design and, hence, in our model there is however, no uncertainty about the stock size, its regeneration rate and carrying capacity as the participants (and agents in the model) are provided with this information.

About 50% of the 19 groups managed the resource cooperatively. Those groups used the opportunity to communicate to make agreements with respect to the exploitation level of the group and all group members followed those agreements during the entire experiment. These agreements were characterized by an equal sharing of the resource. Fig 2 shows the time series of these 19 groups, illustrating the empirical patterns we aim to qualitatively reproduce with AgentEx: there are cooperative (right graph) and non-cooperative (left graph) groups and among both types of groups one can find optimal, over- and underexploitation.

There is a difference between the left and the right graph of Fig 2. Whereas cooperative groups get closer to following the optimal exploitation path by the end of the game, we do not see the same trend in the non-cooperative groups to the left. Nevertheless, for some cooperative groups, it takes almost the whole game to arrive at the MSY even though they have complete knowledge about the resource dynamics; and there is no single group that manages the

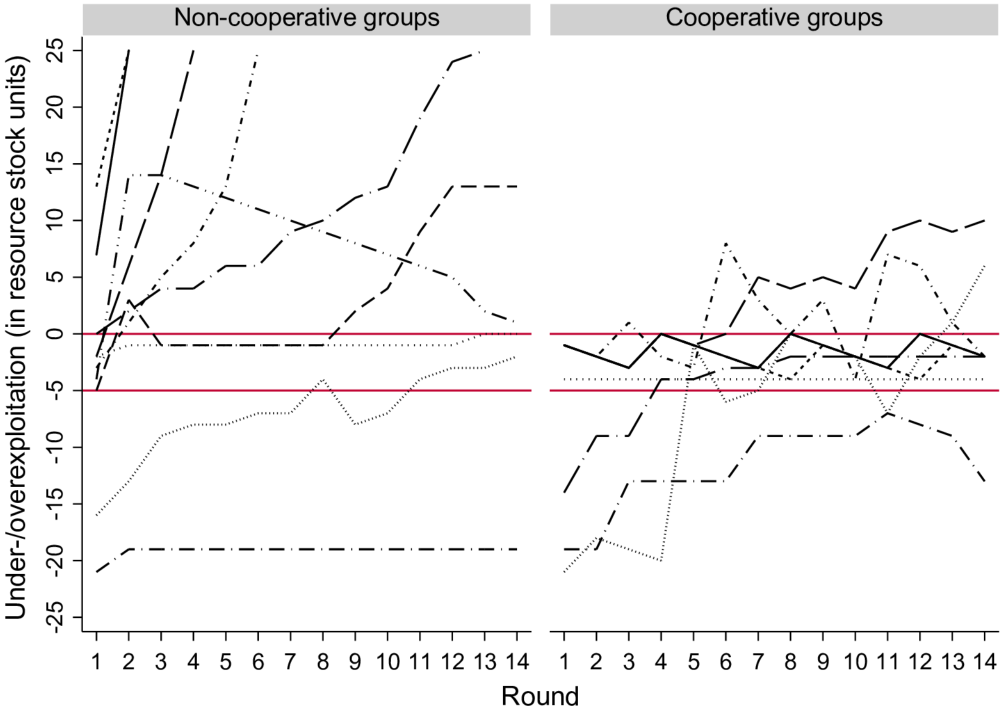

Fig 2. Time Series of Over- and Underexploitation (in Resource Stock Units) for Each Non-Cooperative and Cooperative Group of the Behavioral Experiments. Each line represents one group. Non-cooperative groups (left): N = 10; cooperative groups (right): N = 9. Data points above (below) zero indicate over- (under-) exploitation. The region between the two red lines indicates the MSY. Cooperation is defined as equal sharing of the harvested resource units.

doi:10.1371/journal.pone.0157796.g002

resource optimally during the whole course of the game. Cooperative groups under- and over-exploited the resource in some rounds and a few groups did this throughout the entire game. Thus, cooperation does not lead instantly and automatically to optimal resource use.

We also have data from post-experimental questionnaires on variables such as social skills, trust, fairness and participants' understanding of the resource dynamics and the optimal stock size. In addition, the experimenters observed the participants and kept detailed notes of these observations, which we also made use of for this study. For example, whether or not there were individuals that had a leading role in the development of a group agreement. Each participant gave written consent and could remain anonymous, being identified only through a code; no sensitive data was collected (see S3 Appendix for details on this data).

Towards an Explanation for Cooperative Sustainable Resource Use

The Role of Individual Ecological Knowledge, Confidence in Knowledge and Social Skills

For the purpose of this study, we break down the collective exploitation of a CPR into two major processes: (1) the joint *development of a group agreement on the optimal (sustainable) exploitation level* and (2) the *compliance with the group agreement* (assuming that the group has one). We focus on the first process, as we are interested in what makes cooperative groups manage the CPR sustainably. We argue here that the individual-level attributes: individual knowledge; confidence in knowledge and social skills are the main factors determining the outcome of this process.

The development of a sustainable group agreement requires a) communication and b) knowledge about the ecological dynamics. This knowledge may vary among community and group members and may change over time when users interact with each other (share knowledge) and make experiences with exploiting the CPR, i.e., they learn. A group agreement can only be developed when at least one individual makes use of the opportunity to communicate and shares her ecological knowledge (*individual knowledge*) on what she perceives to be the optimal extraction level.

The behavioral experiments and post-experimental questionnaires revealed that participants varied with respect to their willingness to speak up and share their individual knowledge (see S3 Appendix). We relate and refer to this inter-personal difference in the willingness to communicate and share knowledge as *social skills*. Social skills are often used to describe social behavior of individuals during interpersonal communication in different social contexts and there are many definitions [33]. We define social skills as the ease of an individual to express and discuss her knowledge. This implies that individuals with low social skills are less likely to take part in the formulation of the group agreement. Following, we assume that:

Assumption 1. Speaking up and sharing knowledge is more likely when an individual has high social skills.

The experiment participants that shared their knowledge varied in their influence on the formulation of the group agreement. The experimenters took notes on whether there were participants taking on a leading role in this process. They observed not only that such individuals exist, but also that in case there were two such "leaders" in a group; the one that appeared to be more confident in her knowledge had more influence. To reflect this variety in influence on the formulation of the group agreement, we introduce *confidence in knowledge* in our explanation and use the following assumption:

Assumption 2a. The higher the confidence in knowledge of an individual, the more influence the individual has on the formulation of the group agreement.

The experimenters observed that participants changed their knowledge over time, often influenced by the understanding of other group members. Based on these observations, we hypothesize that confidence in knowledge also plays an important role when an individual updates her knowledge. Previous research has shown that low confidence in one's own knowledge may motivate search for additional information [34] and that high confidence is associated with a lower tendency to acquire new knowledge [35]. Thus we assume:

Assumption 2b. The lower the confidence in knowledge of an individual, the more likely she is influenced by the opinion of others and, hence, updates her knowledge.

Changes in individual perceptions of the optimal stock size can lead to changes in the group knowledge. Such a learning trend on the group level was observed in the exploitation behavior of the experimental participants of some cooperative groups, see Fig 2 (exploitation time series of some cooperative groups move towards the optimal as the experiment progresses). Learning occurs when a resource user reflects on her experiences, for instance whether the outcomes of her actions correspond to her expectations or past experiences [36], or when knowledge is shared amongst resource users through communication or shared activities. If the change of understanding goes beyond the individual and becomes situated within a group social learning takes place [37]. Social learning can lead to improved decision-making, improved problem solving capacities and collective action [38]. In the behavioral experiments and the model, social learning occurs when a group agreement is formed through communication amongst the participants/agents.

Confidence in knowledge can also be interpreted as perceived environmental uncertainty (from the individuals' perspective), i.e., the degree the individual is certain about her own knowledge and feels that she can oversee the consequences of her actions (in our case

extraction). In the case of 'maximum' confidence, the individual is completely certain about her knowledge (in her mind there is no environmental uncertainty). Confidence in knowledge itself can, thus, also be subject to change. It was observed that experiment participants became more (or less) confident during the course of the game. We assume that this is connected to the degree to which the information they received about the state of the resource at the end of each round matched their expectation:

Assumption 3. Confidence in knowledge decreases (increases) when there is a discrepancy (match) between the actual and the expected stock size; the larger the deviation between actual and expected stock size, the stronger the decrease in confidence.

In case of a discrepancy between the actual and the expected stock size, the individual cannot be sure whether this is due to a lack of understanding or because someone took more or less than agreed by the group (for more details see section on 'Description of processes').

From Individual-Level Attributes to Group Outcomes

The experimenters observed that there were instances in which it was sufficient that one group member who was knowledgeable about the optimal extraction level (henceforth called perfect knowledge) spoke up confidently for the group to achieve optimal resource use. Similarly, one individual without perfect knowledge speaking up confidently could lead the group along a non-optimal exploitation path. Based on these observations and the assumptions above, we argue that it is the *distribution* of the individual-level attributes of individual knowledge, confidence in knowledge and social skills within the cooperative group which is crucial for sustainable outcomes. Based on the experimental observations and our explanation, we designed our model analysis to answer the following two questions reflecting different group compositions: i) what difference can (one) informed confident individual(s) make? ii) What effect do opposing individuals, with respect to their knowledge, have?

In a sense, these questions explore the emergence and potential of leaders, or lack thereof, in certain group compositions. However, we are not interested in making a full exploration of all group constellations that could lead to the emergence of leaders and analyze all kinds of leader types. Rather, we want to understand how a certain characteristic of leaders, more precisely their ecological knowledge, may play out in different group settings (i.e., with different distributions of knowledge).

Model Description

The purpose of AgentEx is to qualitatively reproduce and provide an explanation for the patterns observed in the behavioral experiments (see Empirical Background). We are particularly interested in exploring the conditions under which cooperative groups can over- or underexploit the shared resource. The model, hence, follows the experimental setup closely. Fig 3 illustrates the similarity of the setup of the behavioral experiments and AgentEx.

ABMs follow an agent-interaction-environment structure [39,40]. In AgentEx, the agents represent the participants of the behavioral experiments. The environment is represented by the same (abstract) renewable resource as used in the behavioral experiments (Fig 2). The interactions between agents and an agent and the resource are characterized here as: communication/knowledge sharing; the development of a group agreement (social-social interactions) and resource extraction (social-ecological interaction). One time step in the model resembles one round in the behavioral experiment. The simulation runs 14 time steps, resembling the 14 rounds in the experiment.

Our choices on the representation of the agents, their environment and interactions (see assumptions 1–6) are based on the behavioral data as well as on the questionnaire and

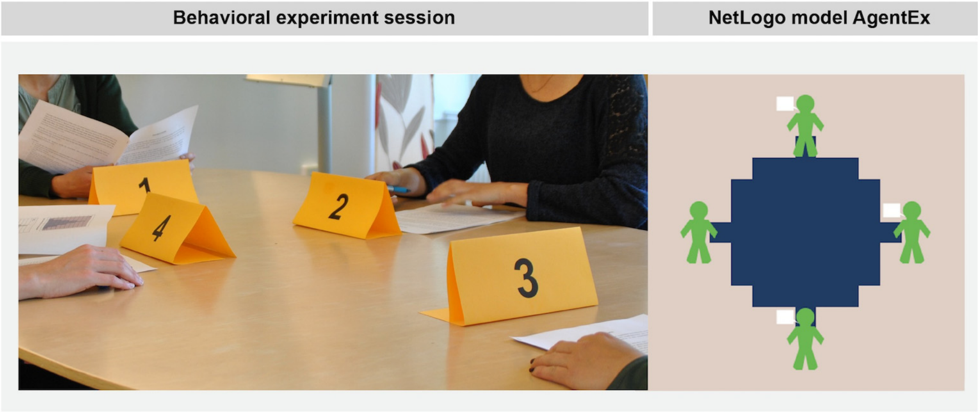

Fig 3. Photograph of a Session of the Behavioral Experiments (Left) and Screenshot of the AgentEx Netlogo Model (Right). Pictures show the similar setup of the behavioral experiments and the AgentEx model. Four individuals/agents share a fictive renewable resource. They have the possibility to communicate, i.e., share knowledge.

doi:10.1371/journal.pone.0157796.g003

observational data from the experimental study and in some cases also informed by literature (e.g., empirical, experimental and theoretical CPR studies, social psychology and communications research). The model was implemented in NetLogo 5.2.0 [41] and is publicly available on openABM (www.openabm.org). A screenshot of the model is provided in Figure F in S2 Appendix and a detailed model description following the ODD+D protocol [42–44] in S1 Appendix.

Agent Attributes

Apart from the three attributes social skills, individual knowledge and confidence in knowledge described above, the agents in AgentEx are characterized by the presence or absence of *social preferences* and their level of *trust* towards the other group members. Trust and social preferences are especially important for the process of compliance with the group agreement (the second process of collective exploitation of a CPR). Table 1 provides definitions and value ranges of the main variables and parameters that characterize the agents, the group and the resource.

Table 1. Overview of Main Variables and Parameters Used in AgentEx.

	Name	Definition	Value range
**Agent**	**Individual knowledge**	The agent's perception of the optimal resource stock size based on its individual understanding of the resource system.	5–50
	**Confidence in knowledge**	The agent's confidence in its knowledge of the resource system.	0.0–1.0
	**Trust**	The agent's belief that the other agents (group members) act as agreed.	0.0–1.0
	**Social skills**[a]	The probability that the agent speaks up, i.e., shares its knowledge with other group members (independent of the group members' actions).	0.0–1.0
	**Social preferences**[a]	The agent has a preference for equal sharing of the resource (or not).	True/False
**Group**	**Group knowledge**	The group's perception of the optimal resource stock size based on the common understanding of the resource system (reflected in the group agreement).	5–50
**Environment**	**Resource stock size**	Stock size in resource stock units.	0–50

Complete set of variables and parameters is provided in S1 Appendix.

[a]These agent attributes do not change over the course of a run.

doi:10.1371/journal.pone.0157796.t001

Numerous experimental studies show that trust and social preferences are important factors for initiating and sustaining cooperation (e.g., [10,45,46]). There are many ways to interpret and define trust and social preferences; in AgentEx an agent is more likely to choose and stick to a group agreement, i.e., cooperate, if it is concerned about a fair distribution of payoffs (social preferences) and if it trusts that the others in the group will do so as well, i.e., social uncertainty is low. Put differently, agents with social preferences can be classified as conditional co-operators [47,48]; they are only willing to cooperate if they think the other group members will do so as well.

Assumption 4. The higher the trust (the lower the social uncertainty), the higher the probability that an agent cooperates.

Some experiment participants took more than agreed upon every once in a while, even within groups characterized by high trust. We explain this non-cooperative behavior in circumstances of high trust as being due to lack of social preferences. In AgentEx, an agent without social preferences is mostly concerned with its own welfare, i.e., to maximize its individual payoff, whereas an agent with social preferences prefers an equal distribution of payoffs. The latter is based on the observation of a strong equal sharing pattern among the cooperative groups in the behavioral experiments [13,14]; and data from the post-experimental questionnaires indicating that fairness played a role in the decision-making for the majority of the participants (see S3 Appendix). These findings are also in line with experimental findings from previous studies (e.g., [49–53]). We thus assume that:

Assumption 5. An agent without social preferences will choose its individual extraction level except when the group extraction level is higher.

In sum, the decision to cooperate (or not) is not linked to a certain 'behavior type' but determined by the interaction of several factors, including social preferences, the level of trust and the expected payoff. For example, social preferences do not automatically lead to cooperation and an agent without social preferences is not automatically a defector.

Whereas social preferences are a constant trait in our model, trust can change over time. Anecdotal observations from the behavioral experiments clearly demonstrated changes in trust. For instance, a drastic decrease in trust occurred when the new stock size did not coincide with what the participants expected. Where the deviation was big, trust eroded to such an extent that in the following round the group depleted the resource. However, when trust in the group was at a high level, cheating did not necessarily lead to depletion.

Assumption 6. The bigger the deviation between expected and actual stock size, the bigger the decrease in trust.

Description of Processes

Fig 4A illustrates our interpretation of the main decision-making processes and actions (in five steps) of participants during one round in the behavioral experiment. We include the explanatory variables (social skills, individual knowledge and confidence) as well as the cooperation facilitators (trust and social preferences) in Fig 4A (in italics and green). These variables represent an influence (outgoing arrows) or are influenced (ingoing arrows) by a given decision-making step. The explanatory variables are critical for step (1), the step we focus on, and trust and social preferences are critical for step (3). Fig 4B illustrates the corresponding implementation of the processes taking place in each time step in the ABM, as briefly outlined below. See Table B in S1 Appendix for a detailed description and formalization of each process.

Given that the agent **communicates**, (A) it shares its knowledge and takes part in **forming the group knowledge** by suggesting a stock size corresponding to its perception of the optimal stock size (individual knowledge). The more confidently the agent shares its knowledge, the

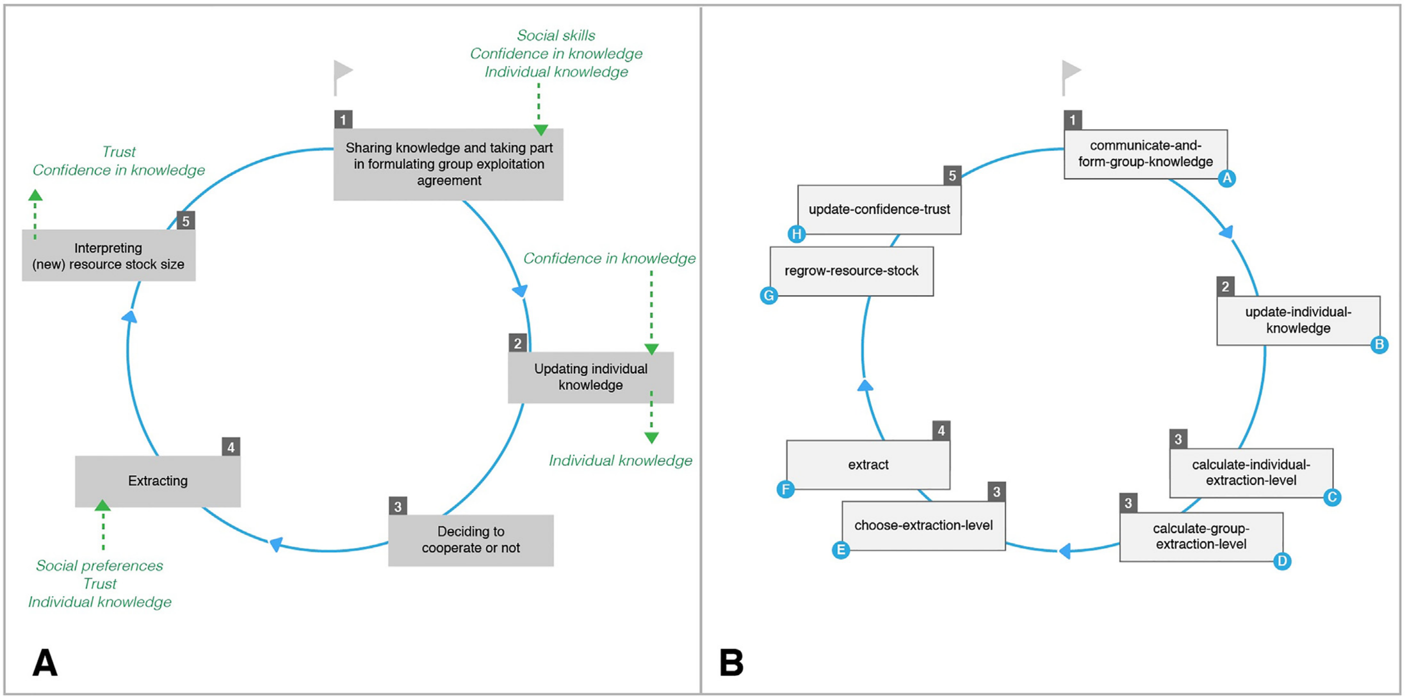

Fig 4. Moving from the Conceptual Model (A) to the Process Diagram (B) of the Model. (A) represents the decision-making processes and actions (in five steps) of an individual during one round of the behavioral CPR experiment. The variables influencing (outgoing arrows) and the variables that are influenced (ingoing arrows) by a given decision-making step are marked in italics and green. (B) represents the process diagram of the model. The numbering in (B) indicates to which step in the decision-making model (A) the processes belong.

doi:10.1371/journal.pone.0157796.g004

more influence it will have on the group knowledge. The group knowledge is determined based on the average of all communicated individual knowledge weighted by the confidence level of each communicating agent. Next (B), the agent might **update** its **individual knowledge** towards the group knowledge depending on its confidence in knowledge and an updating probability. Subsequently (C), the agent **calculates** its **individual extraction level** as the amount of resource units to extract in order to reach the perceived optimal stock size. If the resource stock size is below the perceived optimal size, the agent's extraction level will be zero. If the agent has social preferences, individual extraction will be an equal share of the total amount to be extracted; if it does not have social preferences, the individual extraction level will be higher than the equal share. Likewise, the **group extraction level** (group agreement) is determined as the surplus amount above the optimal stock size reflected in the group knowledge. It is equally shared among all group members.

Once the group and the individual extraction levels are determined, (E) the agent **chooses** its **extraction level** (group or individual) and, hence, to cooperate or not. If the extraction level of the group is higher than the level corresponding to the agent's own individual knowledge, it will choose the group exploitation level. In cases where the group extraction level is lower than the individual level, an agent with social preferences will cooperate if its trust in the group is high. An agent without social preferences will select its individual extraction level.

After (F) **extraction** and (G) **resource regeneration**, (H) the agent receives information about the new resource state. This may lead to an **update of confidence and trust** depending on whether and how the received information matches the agent's expectations. Where the actual stock size is larger than expected (positive deviation), the agent's confidence decreases,

as it attributes this deviation to lack of ecological knowledge. Trust does not change as the agent has no reason to believe that others took more than agreed. Where the new stock size is smaller than expected (negative deviation), both trust and confidence decrease; the agent cannot be sure whether the deviation is because someone took more or because of lack of knowledge. Where there is no deviation, both trust and confidence increase; all agents extracted as per their agreement and the agent was reassured that it understood the resource dynamics correctly.

Simulation Experiments

Building Confidence in the Model

Before using our model to explore the consequences of different group compositions, we verified and validated it [54,55]. We verified the implementation of AgentEx by making sure that it performed according to the logic of our conceptual model (Fig 4A). This included performing a wide exploration of the parameter space through a systematic set of simulations with diverse initial settings and values of the agent attributes (trust, social preferences, social skills, individual knowledge, confidence in knowledge). We tested homogenous as well as heterogeneous group compositions, leading to a total of 6480 unique configurations. See Figure G in S2 Appendix for details (see also Table C in S1 Appendix for the motivation for the initial settings and values chosen and Table D in S2 Appendix for specific definitions of classifications). We repeated each configuration 5000 times to account for the stochasticity in the model (see Calibration and Sensitivity Analysis in S1 Appendix).

We validated the model with this same set of simulations, we also tested our explanation by checking whether the following patterns of the behavioral experiments could be qualitatively reproduced: a) cooperative and non-cooperative outcomes; b) under-, over- and optimal exploitation; c) under-, over- and optimal exploitation outcomes in cooperative groups. This follows the idea of pattern-oriented modelling, where the credibility of a model is assessed based on its ability to reproduce several empirical patterns, which can be considered as "defining characteristics of a system and often, therefore, indicators of essential underlying processes and structures". The aim of this approach is to reduce uncertainty in the model with respect to its structure and parameters [56,57].

Experimental Design to Explore the Impact of Group Composition

To explore the impact of group composition, we focused on configurations that are classified as cooperative (see Figure G in S2 Appendix) and developed seven different scenarios (group compositions), divided into two sets, see Fig 5. The sets were guided by the two questions presented above: ii) What difference can (one) informed, confident agent(s) make? (Scenario set I) and ii) What effect do agents with opposing knowledges have? (Scenario set II).

We varied the group compositions by manipulating the three explanatory variables (individual knowledge, confidence in knowledge and social skills) in the following way: The agents in a group were divided into "informed" and "uninformed" agents. Informed agents are initialized with perfect knowledge. They thus perceive the optimal stock size to be between 25 and 29, the range of the MSY. The knowledge of the uninformed agents is initialized to reflect overexploitation, i.e., resource stock size between 10 and 24 (see Fig 1). Moreover, agents either speak and share their knowledge with the others (social skills = 1), or not (social skills = 0) and their confidence in knowledge is either high (0.8) or low (0.2).

Since we are interested in what it takes to optimally manage the CPR for cooperative groups, we set the initial settings as follows: a minimum of one agent shares its knowledge (to allow group knowledge); all agents in all scenarios had social preferences and were initialized with

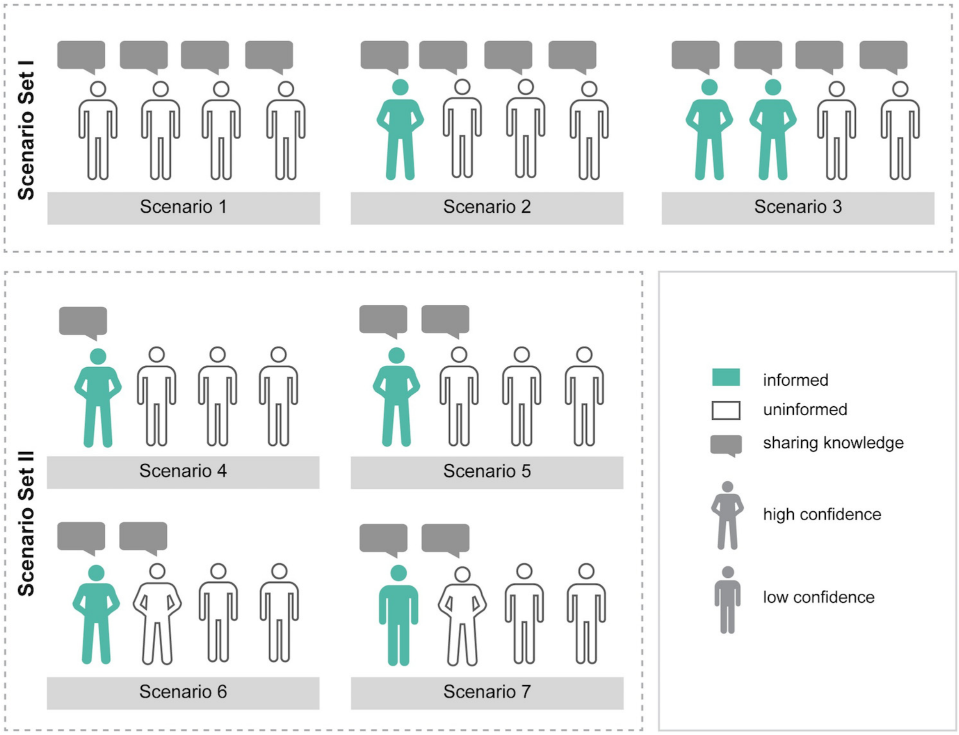

Fig 5. Visual Description of Developed Scenarios in Two Sets to Explore the Impact of Group Composition. (Not) sharing knowledge implies that social skills are set to a value of 1 (0). Informed agents are initialized with an individual knowledge value between 25 and 29 and uninformed agents between 5 and 24 respectively. High (low) confidence implies an initial value of 0.8 (0.2). Non-speakers always have low confidence and are uninformed to ensure tractability. Agents that do not speak up and share will not influence the group knowledge. In all scenarios, all agents have social preferences and are initialized with high trust values (0.66 to 0.94). See Table 1 and Table C in S1 Appendix for details.

doi:10.1371/journal.pone.0157796.g005

high trust values (between 0.66 and 0.94), i.e., the basic conditions for cooperation we identified in the simulation experiment to build confidence in our model. See Fig 5 for details of the manipulations.

Scenario set I. To explore the effect of (one) confident informed agent(s) on a group of uninformed agents (scenario 1–3), all agents share their knowledge and only individual knowledge and confidence in knowledge vary. There are only uninformed agents with low confidence in scenario 1; in scenario 2 we change one uninformed low-confidence agent to an informed one with high confidence and in scenario 3, another informed high-confidence agent is added. By varying the number of agents with high and low confidence, we also vary the overall level of perceived environmental uncertainty in the group. For example, in scenario 1 all agents have low confidence, representing a case with a high level of perceived environmental uncertainty. This scenario can then be contrasted with scenario 2 and 3, where the level of perceived environmental uncertainty is lower.

Scenario set II. In set II (scenario 4–7), individual knowledge is constant: three agents are uninformed and one agent is informed. We varied the level of confidence as well as the number of agents sharing their knowledge (one in scenario 4 and two in the other three scenarios). The informed agent always shares its knowledge and is confident in scenarios 4–6 and not confident in scenario 7. The confidence of the uninformed speaking agent in scenarios 5, 6 and 7 also differs. It is low in scenario 5 and high in scenario 6 and 7. In this way, we could explore

the effect of an uninformed speaking agent (with low or high confidence) opposing the optimal extraction level of the informed speaking agent (with low or high confidence). Moreover, since the scenarios in set II all have the same overall level of individual knowledge but differ with respect to number and distribution of speakers and the overall level and distribution of perceived environmental uncertainty, we can assess how these attributes influence the outcome. For example, scenario 6 and 7 differ only in the level of perceived environmental uncertainty in the group.

We are also interested to see how confidence in knowledge, i.e., the perceived environmental uncertainty, may or may not interact with social uncertainty and if this potential interplay affects the likelihood for the group to manage the resource optimally. Therefore, in the final step, we also explore the robustness of cooperative optimal exploitation to increased social uncertainty. For that purpose, we introduced an upper limit for the initial trust range for all seven scenarios. Instead of allowing for the whole range between 0.66 to 0.94, we limited the upper bound to 0.8, see Table C S1 Appendix for details on initial value settings.

Results

Model Evaluation

The results of the simulation experiment to build confidence in the model verify, not surprisingly, that trust and social preferences are crucial for cooperative outcomes. In all configurations that are classified as cooperative, all agents start with high initial trust values and all have social preferences. See Figure G in S2 Appendix for how the various initial setting combinations relate to the outcome patterns. The macro-level outcomes such as cooperation, under-, over- and optimal exploitation, resulting from the broad screening of initial conditions and parameter settings corresponds well with the macro-level outcomes of the behavioral experiments. There are configurations that lead to cooperative and non-cooperative outcomes and all three exploitation patterns (optimal, over- and under-exploitation; see Table D in S2 Appendix for definition of classifications) are represented.

Fig 6 compares the exploitation patterns between cooperative groups from the behavioral experiments (left graph) with unique model runs randomly drawn from configurations that lead to cooperation and in which all agents chose the group exploitation level in each time step (right graph). The two graphs indeed show similar qualitative patterns. For example, as in the behavioral experiments, there are cooperative runs in which the agents over- and underexploit the resource during some ticks or the whole course of the run. But there are also runs in which the agents achieve the MSY throughout the run. Moreover, in both graphs some time series are characterized by a learning trend, the exploitation path of some groups/runs move closer to optimal towards the end of the experiment/run. We can thus confirm that the explanation based on the interactions of individual ecological knowledge, confidence in knowledge and social skills is a plausible explanation of the outcomes of the behavioral experiments.

Group Composition Matters

Scenario set I: The difference (one) informed confident agent(s) can make. The difference that one informed confident agent can make for a cooperative group to manage the CPR optimally becomes apparent when comparing the resource stock size graphs of scenario 1 and 2 (Fig 7). It shows that such an agent will stimulate its uninformed group members to pursue a path that is considerably closer to optimal exploitation even if this results in lower payoffs for each agent. Note that the overall level of lack of knowledge is not much lower in scenario 2 compared to scenario 1. This highlights the importance of the distribution (in addition to absolute levels) of knowledge and confidence in knowledge for the development of the group

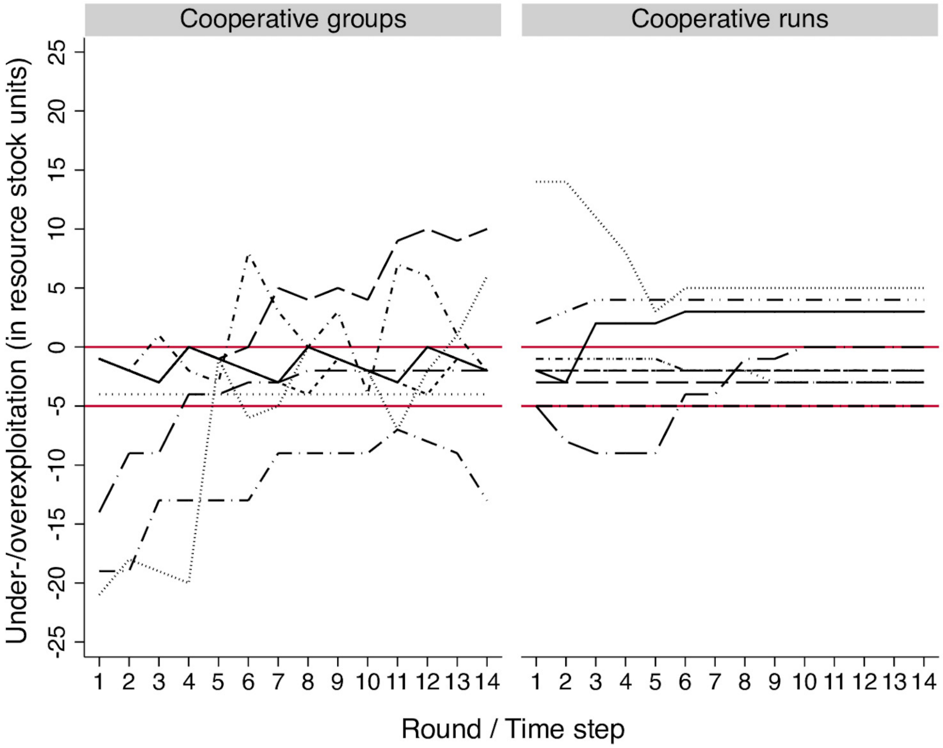

Fig 6. Comparison of Cooperative Exploitation Patterns in the Behavioral Experiments (Left) and AgentEx (Right). Each line represents one group/run. Cooperative groups/runs: N = 9/10. Data points above (below) zero indicate over- (under-) exploitation. The region between the two red lines indicates the MSY. Cooperation is here defined as equal sharing of the harvested resource units.

doi:10.1371/journal.pone.0157796.g006

extraction level. The more informed agents with high confidence, the stronger the increase towards optimal exploitation (compare scenario 3 with 2 and 1, Fig 7). If no one is confident and the average knowledge is low, this group will inevitably overexploit (scenario 1, Fig 7).

The difference in the outcomes can be explained by the willingness of the other group members to *change* their knowledge. This willingness is determined by the level of confidence. If those agents whose ecological knowledge is incorrect are willing to change their knowledge, the group can approach the sustainable group extraction level. If the uninformed agents were more confident, this effect would immediately diminish, as the agents would be less susceptible to change.

The specific mechanism behind this outcome is the following: as all group members communicate, they all influence the group knowledge. However, the knowledge of the confident agent will weigh in more. The resulting group knowledge will be slightly below the optimal stock size. The uninformed agents will repeatedly update their individual knowledge towards the group knowledge because of their low confidence. Their confidence will also increase over time (because the feedback they receive from the resource confirms their expectations). However, the influence of the informed confident agent is stronger throughout the entire run. This makes the group knowledge level less susceptible to change (see Figure F in S2 Appendix for details on this mechanism illustrated with a screenshot of scenario 2).

Scenario set II: The impact opposing agents can have. Sharing knowledge and being informed and confident has a positive effect on the exploitation path in a group of uninformed, low-confidence agents (compare scenario 2 in Fig 7 with scenario 4 in Fig 8). However, by

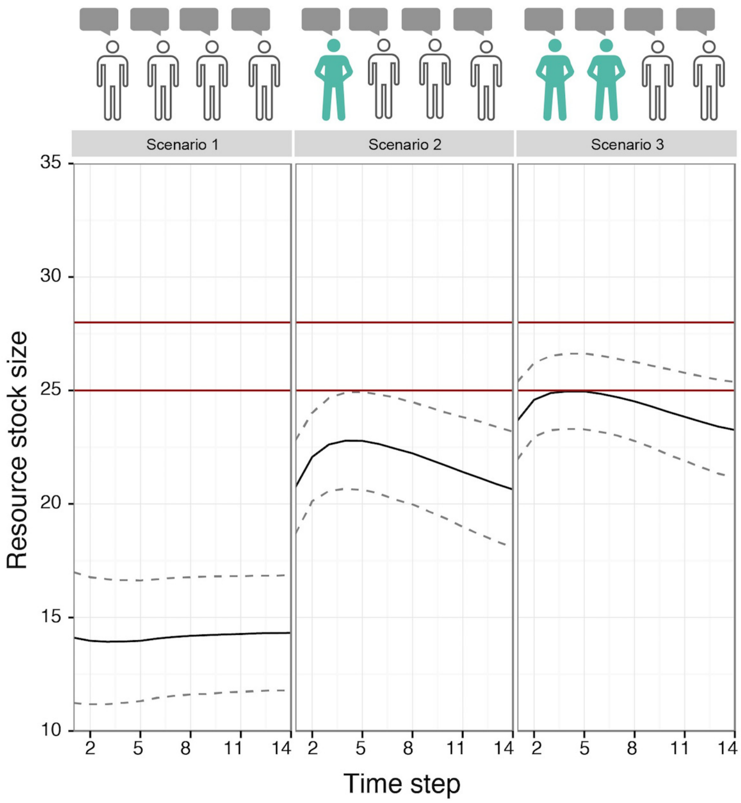

Fig 7. The Influence of (One) Informed Confident Agent(s) on the Group Exploitation Pattern (Scenario Set I). The graphs illustrate the average resource stock size per scenario. The range between the red lines indicates optimal exploitation (in the sense that the MSY is achieved). The agent icons illustrate the scenario settings: sharing knowledge (speaking bubble), being informed (green body), being confident (confident position); see Fig 5. Note that all scenarios lead to cooperative outcomes. The downward trend of the resource stock size curve in scenario 1 and 2 is due to the increasing influence of the uninformed agents on group knowledge over time, as they become more confident.

doi:10.1371/journal.pone.0157796.g007

adding one other confident, but uninformed, speaking agent, this positive effect weakens (compare scenario 5 with 6). The gradual effect of this 'weakening', which leads to deviation from the optimal group exploitation is visible in Fig 8, starting from scenario 4. Two speakers one of whom is confident and informed and the other uninformed, but not confident, already weakens the effect a little (scenario 5, Fig 8). A confident opposing speaker (scenario 6) cancels out the change towards optimal behavior. Scenario 7 takes it to the most non-optimal extreme, making the one that is informed not confident, opposed to a confident uninformed agent.

Robustness of Cooperative Optimal Exploitation to Increased Social Uncertainty

Having explored conditions for cooperative optimal exploitation, where agents have different perceptions of environmental uncertainty, one last question remains: how robust is this cooperative optimal state to social uncertainty? Until this point, we focused on cooperative groups and therefore, kept social uncertainty low and constant. Social uncertainty relates to trust, i.e., the perception of the agents about whether or not the other ones are sticking to the group agreement. We now explore the role of these conditions under increased social uncertainty.

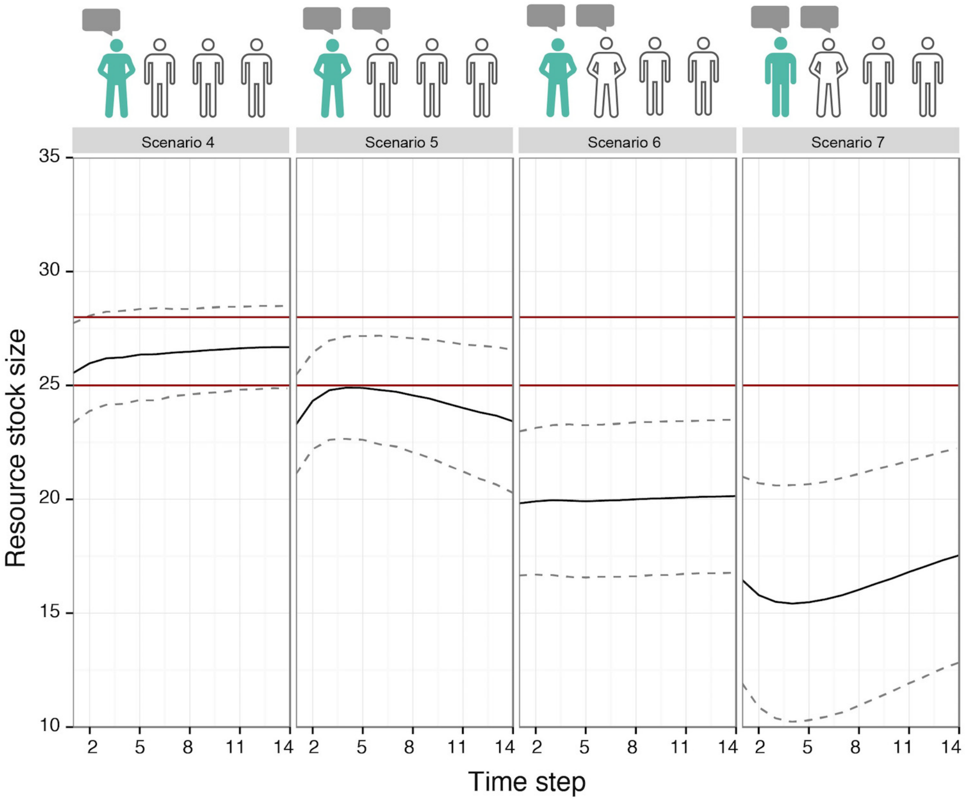

Fig 8. The Influence of Agent(s) with Opposing Knowledges on the Group Exploitation Pattern (Scenario Set II). The graphs illustrate the average resource stock size per scenario. The range between the red lines indicates optimal exploitation (in the sense that the MSY is achieved). The agent icons illustrate the scenario settings: sharing knowledge (speaking bubble), being informed (green body), being confident (confident position); see Fig 5. Note, all scenarios lead to cooperative outcomes. The downward trend of the resource stock size curve in scenario 5 is due to the increasing influence of the uninformed agent on group knowledge over time, as it becomes more confident.

doi:10.1371/journal.pone.0157796.g008

The results shows that increased social uncertainty does not have an effect on the *exploitation patterns* of the seven scenarios. There are, in fact, only minimal differences in the exploitation patterns. However, higher social uncertainty makes some scenarios result in non-cooperative outcomes. While there is no effect for the scenarios of scenario set I, two scenarios (scenario 4 and 5) of scenario set II do not classify as cooperative any longer. The scenarios that continue to show cooperative outcomes under increased social uncertainty are characterized by one of the following three properties: i) speaking, uninformed agents have equal or higher confidence than speaking informed agents. This results in a lower discrepancy between group knowledge and individual knowledge (scenario 6 and 7), which in turn reduces the probability that the uninformed agents choose their individual extraction level and, hence, do not cooperate. ii) Two informed, speaking agents with high confidence (scenario 3). This reduces the number of uninformed agents that could opt for the individual extraction level; or iii) all agents speak and there is a minimum of three uninformed agents in the group (scenario 1 and 2). In this case, there is no or very low discrepancy between group and individual knowledge making it very unlikely for the uninformed agents to choose their individual extraction level. Considering our interest in conditions for sustainable collective management, case ii) is particularly interesting because this scenario leads to cooperative and close to optimal exploitation even under increased social uncertainty.

Overall, this robustness test shows that cooperation, in comparison to optimal exploitation, is more sensitive to increased social uncertainty. While optimal exploitation is robust towards increased social uncertainty, the loss of cooperation results in an unequal distribution of earnings. The initial settings of social uncertainty/trust have such a strong influence on cooperation because they determine whether or not an agent chooses its own individual extraction level over the group extraction level when the individual extraction level yields higher payoffs. Hence, the lower the initial trust values, the harder it will be for cooperation to emerge when the group contains uninformed agents. The erosion of trust might be less strong when the non-speaking agents also have perfect knowledge.

Discussion and Conclusions

Recent observations in behavioral CPR experiments show that cooperation does not necessarily imply sustainable (optimal) resource use [13,14]. In this paper, we proposed a social-ecological explanation for these observations. It specifies how individual-level and group attributes interplay within a dynamic social-ecological environment to produce outcomes where cooperation goes hand-in-hand with sustainable CPR use. In the development of the explanation, we followed a mechanism-based approach, i.e., an approach that details the "cogs and wheels of the causal process through which the outcome to be explained was brought about" (see page 50 in [23]). To this end, we first developed a hypothesis about relevant micro-level mechanisms that may explain the outcomes of the behavioral experiments based on the insights of the behavioral study as well as insights from previous research. We then formalized our hypothesis in an ABM and simulated the resulting macro-level outcomes. The macro-level model outcomes correspond well with the observed exploitation patterns, such as over-, under- and optimal resource exploitation. We can thus confirm the validity of our explanation as one possible mechanism-based explanation for the non-sustainable cooperative resource use in self-governed CPR settings. Note that there are other possible explanations given that we are dealing with a complex social-ecological system. However, we believe that our explanation highlights critical factors and interactions for sustainable resource use, namely ecological knowledge, confidence in knowledge and social skills. Subsequent work will focus on testing competing mechanisms.

Our explanation is characterized by three individual-level variables: individual ecological knowledge, confidence in knowledge and social skills that interact to determine individual and group knowledge as the outcomes of communication/knowledge sharing and knowledge updating (learning). The processes of individual and social learning have proved to be key mechanisms for sustainable cooperative resource use under social and ecological uncertainty [38]. The development of the individual as well as the shared understanding of the optimal extraction level does, however, depend on the distribution of the three variables within and across agents. It is, for instance, not sufficient for cooperative sustainable resource use that the group has a 'good' average ecological knowledge (optimal stock size). The distribution of this ecological knowledge within the group, in combination with the individual's confidence in knowledge and the willingness of each individual to share her knowledge with the other group members (i.e., social skills) are also critical determinants for sustainable outcomes.

We show that there are multiple group constellations that can lead to (close to) optimal exploitation. At the group level, good group knowledge is needed. 'Good' group knowledge can be achieved, for example, by a group of agents most of which are uninformed (and silent) with low confidence, but at least one of them is informed and confident. These findings highlight the importance of considering not only the heterogeneity of attributes between people (intra-personal), but also the combination of these attributes within a person (inter-personal).

Confidence in knowledge represents perceived environmental uncertainty (from the individual's perspective). The effect of environmental uncertainty in CPR games has been studied before. For example, some experimental studies in social psychology have investigated how resource users in commons dilemmas react to uncertainty regarding resource stock size and/or regeneration rate [58]. These studies show that experiment participants are more prone to overexploit the CPR in the face of environmental uncertainty [59–62]. However, these studies rest on the assumption of an equal distribution of perceived environmental uncertainty in the group. We show that it is crucial to explore the *heterogeneity* of *perceived* environmental uncertainty and other individual attributes. Our research demonstrates that (perceived) environmental uncertainty is not necessarily bad, opposing their findings; it can serve as an incentive to learn and find a good exploitation path. The results described above let us conclude that the higher uninformed agents' perception of environmental uncertainty (aka the lower their confidence), the more likely the group is to achieve optimal exploitation provided that there is at least one informed agent who shares its knowledge.

AgentEx was developed to identify micro-foundations underlying the exploitation patterns observed in the behavioral experiments of [13,14]. Using an agent-based modeling approach grounded in the insights of this behavioral work allowed us to manipulate, control and measure factors internal to a person and analyze interactions between these factors, on the individual and group level as well as between the individuals and the resource (social-ecological feedbacks). The exercise of building the ABM helped us clarify and specify elements of our explanation, particular critical social and social-ecological interactions considered critical for cooperative sustainable resource use beyond the social and institutional realm (e.g., ecological knowledge and confidence in knowledge). In the future, we can use AgentEx to test specific alternative hypotheses about individual and collective decision-making in commons dilemmas with explicit social-ecological feedbacks.

The behavioral experiments and the model are both simplifications of real-world decision-making processes and contexts. They cannot be used directly to develop real-world policies or management recommendations. However, the mechanism-based understanding gained from this exercise offers valuable insights for policy-making in the context of community-based management of CPR. These include that a) not every member of a resource user community needs to have perfect ecological knowledge in order for the community to secure the long-term provision of the CPR given that there exist processes where sharing of knowledge and experiences is possible; b) knowledge sharing is crucial and c) perceived environmental uncertainty is not necessarily a bad thing, as it can open up sensitivity for change and possibilities for learning. The interpretation of ecological feedback that leads to changes in individual understanding, and the sharing of this knowledge through communication and deliberation within a group of resource users, where this understanding becomes situated and leads to a group agreement, are both important elements of social learning [37]. We assume that individual learning is stronger in individuals that perceive their ecological environment as uncertain. This individual learning contributes to the social learning in the group, given that the individual participates in the deliberation process. The outcomes of the social learning process that takes place during the development of the group exploitation agreement depend on the distribution and interaction of knowledge, confidence in that knowledge and social skills. Learning can thus lead to improved resource use if there is a leader with the adequate ecological knowledge.

Many empirical CPR studies emphasize the importance of leadership for successful management [63,64]. In the experimental literature on social dilemmas, there is one strand trying to understand when leaders are more likely to emerge. Some argue, for example, that leadership is more likely to emerge when coordination is necessary (see, e.g., [65]). Others have found that groups are more willing to appoint a leader when they feel they cannot manage the resource

(have experienced past failures) or when they feel that past distributions of benefits have been unequal (see, e.g., series of experiments by [66,67]). In our model, leadership is not the consequence of a deliberate process, such as appointing somebody, but emerges from the distribution of individual attributes. There will only be a leader in the group if there is at least someone who has the social skills to speak up and share her knowledge and if that person's confidence in her knowledge is high enough *relative* to the confidence of the other group members. Our model, thus, also allows and can explain leadership associated with poor resource management.

Our model can, of course, be enriched and modified further and thereby allow for a variety of additional explorations. For example, group knowledge formation, i.e., the process for how individuals share their individual knowledge and develop a shared understanding, features prominently in our explanation. There are many ways this process of social learning can take place [68] and, hence, a natural next step would be to test other ways of forming group knowledge. Another process to pay attention to is the connection between trust and communication. In our model, trust is only influenced by whether or not agents act as agreed. However, communication is often said to promote trust in groups (see, e.g., [19]). One could think, hence, of linking trust to communication by introducing a positive feedback between both. This way, agents could start with a lower trust value and still decide to cooperate at a later stage. Moreover, we have assumed that social preferences are context independent, i.e., that an agent without social preferences always chooses its individual extraction level, unless the group extraction level pays off more (no matter the trust in the group). There is, however, evidence that social preferences can also be determined (at least partially) by the (social) context. Knowledge from, for example, environmental psychology could be a source for refining this aspect of AgentEx, e.g., [9].

For this paper's purpose, we kept a fairly simple description of the ecological system but for another purpose it may be fruitful to incorporate more realistic ecosystem dynamics. For example, in our model there is no 'external' uncertainty imposed and the dynamics are fairly 'smooth'. It would be interesting to allow for more abrupt changes, so-called regime shifts [69,70], and account for their inherent uncertainties (e.g., probability of there being a regime shift, location of regime shifts). In the face of such ecological changes and uncertainties, confident individuals (leaders), knowledgeable about the ecological dynamics might be yet more crucial.

The study provides insights about critical social-ecological mechanisms underpinning human behavior, collective action and sustainable resource management, which can inform case-study research, as these mechanisms are crucial to understanding the micro-foundations and dynamics of local social-ecological systems. One of the next steps for AgentEx is to connect back to behavioral experiments in the lab and in the field as well as case study research for guidance about future model extensions and interpretations. This would also allow us to test whether the here-developed explanation is valid beyond the experimental lab.

Supporting Information

S1 Appendix. Model Description. ODD+D protocol, details on model process implementation, calibration and sensitivity analysis.
(PDF)

S2 Appendix. Supplementary Information on Simulation Experiments.
(PDF)

S3 Appendix. on Behavioral Experiments Data.
(PDF)

S1 Data. Building Confidence in Supplementary Information Model Simulation Experiment.
(CSV)

S2 Data. Scenarios Simulation Experiment.
(CSV)

Acknowledgments

We are grateful to Stockholm Resilience Centre and the Beijer Institute of Ecological Economics (through the Kjell and Märta Beijer Foundation) for facilitating and supporting our workshops that created the space for the development of AgentEx. We are also very grateful to Örjan Bodin, Elin Enfors, Peter Martinsson, Geeske Scholz, Björn Vollan and an anonymous reviewer for feedback and comments on earlier versions of this paper. A special thanks also to Ana Ospina Medina for graphical support. All remaining errors are ours.

Author Contributions

Conceived and designed the experiments: CS NW MS TL.

Performed the experiments: NW CS.

Analyzed the data: CS NW MS TL.

Wrote the paper: CS NW MS TL.

Developed the model: NW CS MS TL.

References

1. Hardin G. The Tragedy of the Commons. Science. 1968; 162(3859):1243–8.
2. Dawes RM. Social dilemmas. Annu Rev Psychol. 1980; 31:169–93.
3. Berkes F. Common Property Resources: Ecology and Community-based Sustainable Development. London: Belhaven Press; 1989.
4. Ostrom E. Governing the Commons: The Evolution of Institutions for Collective Action. New York: Cambridge University Press; 1990.
5. Ostrom E, Dietz T, Dolšak N, Stern PC, Stonich S, Weber EU. The Drama of the Commons. Washington, DC: National Academy Press; 2002.
6. Wade R. Village Republics: Economic Conditions for Collective Action in South India. Cambridge, UK: Cambridge Univeristy Press; 1988.
7. Hanna SS, Folke C, Mäler K-G. Rights to nature: cultural, economic, political, and economic principles of institutions for the environment. Washington, DC: Island Press; 1996.
8. Bromley DW, Feeny D, McKean MA, Peters P, Gilles JL, Oakerson RJ, et al. Making the Commons work: Theory, Practice, and Policy. San Francisco: ICS Press; 1992.
9. Biel A, Eek D, Gärling T, Gustafsson M. New Issues and Paradigms in Research on Social Dilemmas. New York: Springer; 2008.
10. Ostrom E. A Behavioral Approach to the Rational Choice Theory of Collective Action: Presidential Address, American Political Science Association, 1997. Am Polit Sci Rev. 1998; 92(1):1–22.
11. Walters CJ, Martell SJ. Fisheries ecology and management. New York: Princeton University Press; 2004.
12. Berkes F, Folke C. Linking Social and Ecological Systems: Management Practices and Social Mechanisms for Building Resilience. Cambridge, UK: Cambridge Univeristy Press; 1998.

13. Schill C, Lindahl T, Crépin A-S. Collective action and the risk of ecosystem regime shifts: insights from a laboratory experiment. Ecol Soc. 2015; 20(1):48.
14. Lindahl T, Crépin A-S, Schill C. Potential Disasters can Turn the Tragedy into Success. Environ Resour Econ. First online. doi: 10.1007/s10640-016-0043-1
15. Ostrom E. The value-added of laboratory experiments for the study of institutions and common-pool resources. J Econ Behav Organ Organ. 2006; 61(2):149–63.
16. Balliet D. Communication and Cooperation in Social Dilemmas: A Meta-Analytic Review. J Conflict Resolut. 2009; 54(1):39–57.
17. Sally D. Conversation and Cooperation in Social Dilemmas: A Meta-Analysis of Experiments from 1958 to 1992. Ration Soc. 1995; 7(1):58–92.
18. Pretty J. Social capital and the collective management of resources. Science. 2003; 302(5652):1912–4. PMID: 14671287
19. Shankar A, Pavitt C. Resource and Public Goods Dilemmas: A New Issue for Communication Research. Rev Commun. 2002; 2.3:251–72.
20. Poteete AR, Janssen MA, Ostrom E. Working Together: Collective Action, the Commons, and Multiple Methods in Practice. Princeton, NJ: Princeton University Press; 2010.
21. Walters CJ. Adaptive Management of Renewable Resources. New York: Macmillan; 1986.
22. Hedström P, Ylikoski P. Causal Mechanisms in the Social Sciences. Annu Rev Sociol. 2010; 36:49–67.
23. Miller JH, Page SE. Complex Adaptive Systems: An Introduction to Computational Models of Social Life. Princeton, NJ: Princeton University Press; 2007.
24. Bruch E, Atwell J. Agent-Based Models in Empirical Social Research. Sociol Methods Res. 2015; 44 (2):186–221. PMID: 25983351
25. Le Page C, Bazile D, Becu N, Bommel P, Etienne M, Mathevet R, et al. Agent-Based Modelling and Simulation Applied to Environmental Management. In: Edmonds B, Meyer R, editors. Simulating Social Complexity. Berlin, Heidelberg: Springer; 2013. p. 499–540.
26. Bodin O, Norberg J. Information network topologies for enhanced local adaptive management. Environ Manage. 2005; 35(2):175–93. PMID: 15902456
27. Schlüter M, Pahl-Wostl C. Mechanisms of Resilience in Common-pool Resource Management Systems: an Agent-based Model of Water Use in a River Basin. Ecol Soc. 2007; 12(2):4.
28. Pérez I, Janssen MA. The effect of spatial heterogeneity and mobility on the performance of social–ecological systems. Ecol Modell. 2015; 296:1–11.
29. Deadman PJ, Schlager E, Gimblett R. Simulating Common Pool Resource Management Experiments with Adaptive Agents Employing Alternate Communication Routines. J Artif Soc Soc Simul. 2000; 3(2).
30. Janssen MA. An Agent-Based Model Based on Field Experiments. In: Smajgl A, Barreteau O, editors. Empirical Agent-Based Modelling—Challenges and Solutions: Volume 1: The Characterisation and Parameterisation of Empirical Agent-Based Models. New York: Springer; 2014. p. 189–205.
31. Janssen MA, Rollins N. Predicting Behavior in New Behavioral Experiments: Outcomes of a Modeling Competition. J Artif Soc Soc Simul. 2012; 15(3):5.
32. Ludwig D, Hilborn R, Walters C. Uncertainty, Resource Exploitation, and Conservation: Lessons from History. Science. 1993; 260(5104):17+36. PMID: 17793516
33. Hargie O, Sanders C, Dickson D. Social skills in interpersonal communication. London: Routledge; 1994.
34. Chaiken S, Liberman A, Eagly AH. Heuristic and systematic information processing within and beyond the persuasion context. In: Uleman JS, Bargh JA, editors. Unintended thought. New York: Guilford; 1989. p. 212–52.
35. Böhm G, Pfister H-R. Mental representation of global environmental risks. Res Soc Probl Public Policy. 2001; 9:1–30.
36. Fazey I, Fazey JA, Fazey DMA. Learning More Effectively from Experience. Ecol Soc. 2005; 10(2):4.
37. Reed M, Evely A, Cundill G, Fazey I, Glass J, Laing A, et al. What is Social Learning? Ecol Soc. 2010; 15(4):r1.
38. Cundill G, Rodela R. A review of assertions about the processes and outcomes of social learning in natural resource management. J Environ Manage. 2012; 113:7–14. doi: 10.1016/j.jenvman.2012.08.021 PMID: 22985710
39. Gilbert N. Agent-based models. Thousand Oaks, CA: Sage; 2008.
40. Edmonds B, Meyer R. Simulating Social Complexity. A Handbook. Berlin, Heidelberg: Springer; 2013.

41. Wilensky U. NetLogo Center for Connected Learning and Computer-Based Modeling. Evanston, IL: Northwestern University; 1999.
42. Grimm V, Berger U, DeAngelis DL, Polhill JG, Giske J, Railsback SF. The ODD protocol: A review and first update. Ecol Modell. 2010; 221(23):2760–8.
43. Müller B, Bohn F, Dreßler G, Groeneveld J, Klassert C, Martin R, et al. Describing human decisions in agent-based models–ODD + D, an extension of the ODD protocol. Environ Model Softw. 2013 Oct; 48:37–48.
44. Grimm V, Berger U, Bastiansen F, Eliassen S, Ginot V, Giske J, et al. A standard protocol for describing individual-based and agent-based models. Ecol Modell. 2006; 198(1–2):115–26.
45. Fehr E. On The Economics and Biology of Trust. J Eur Econ Assoc. 2009; 7(2–3):235–66.
46. Fehr E, Fischbacher U. Why social preferences matter—the impact of nonselfish motives on competition, cooperation, and incentives. Econ J. 2002; 112:C1–33.
47. Fischbacher U, Gächter S, Fehr E. Are people conditionally cooperative? Evidence from a public goods experiment. Econ Lett. 2001; 71(3):397–404.
48. Kocher MG, Cherry T, Kroll S, Netzer RJ, Sutter M. Conditional cooperation on three continents. Econ Lett. 2008; 101(3):175–8.
49. Allison ST, McQueen LR, Schaerfl LM. Social Decision Making Processes and the Equal Partitionment of Shared Resources. J Exp Soc Psychol. 1992; 28(1):23–42.
50. Fehr E, Schmidt KM. A theory of fairness, competition, and cooperation. Q J Econ. 1999; 114(3):817–68.
51. De Kwaadsteniet EW, Van Dijk E, Wit A, De Cremer D. Social dilemmas as strong versus weak situations: Social value orientations and tacit coordination under resource size uncertainty. J Exp Soc Psychol. 2006; 42(4):509–16.
52. Lindahl T, Johannesson M. Bargaining over a Common Good with Private Information. Scand J Econ. 2009; 111(3):547–65.
53. Tricomi E, Rangel A, Camerer CF, O'Doherty JP. Neural evidence for inequality-averse social preferences. Nature. 2010; 463(7284):1089–91. doi: 10.1038/nature08785 PMID: 20182511
54. David N. Validating Simulations. In: Edmonds B, Meyer R, editors. Simulating Social Complexity. Berlin, Heidelberg: Springer; 2013. p. 135–71.
55. Balci O. Golden Rules of Verification, Validation, Testing, and Certification of Modeling and Simulation Applications. SCS M&S Mag. 2010; 4(4):1–7.
56. Grimm V, Revilla E, Berger U, Jeltsch F, Mooij WM, Railsback SF, et al. Pattern-Oriented Modeling of Agent-Based Complex Systems: Lessons from Ecology. Science. 2005; 310(5750):987–91. PMID: 16284171
57. Janssen MA, Radtke NP, Lee A. Pattern-Oriented Modeling of Commons Dilemma Experiments. Adapt Behav. 2009; 17(6):508–23.
58. Messick DM, Allison ST, Samuelson CD. Framing and communication effects on group members responses to environmental and social uncertainty. In: Maital S, editor. Applied behavioral economics. New York: New York University Press; 1988. p. 677–700.
59. Budescu D V., Rapoport A, Suleiman R. Resource dilemmas with environmental uncertainty and asymetric players. Eur J Soc Psychol. 1990; 20:475–87.
60. Rapoport A, Budescu D V., Suleiman R, Weg E. Social dilemmas with uniformly distributed resources. In: Liebrand WBG, Messick DM, Wilke HAM, editors. Social dilemmas: Theoretical issues and research findings. Oxford, UK: Pergamon Press; 1992. p. 43–57.
61. Gustafsson M, Biel A, Gärling T. Overharvesting of resources of unknown size. Acta Psychol (Amst). 1999; 103:47–64.
62. Hine DW, Gifford R. Individual Restraint and Group Efficiency in Commons Dilemmas: The Effects of Two Types of Environmental Uncertainty. Jounral Appl Soc Psychol. 1996; 26(11):993–1009.
63. Gutiérrez NL, Hilborn R, Defeo O. Leadership, social capital and incentives promote successful fisheries. Nature. 2011; 470(7334):386–9. doi: 10.1038/nature09689 PMID: 21209616
64. Agrawal A. Common Resources and Institutional Sustainability. In: Ostrom E, Dietz T, Dolšak N, Stern PC, Stonich S, Weber EU, editors. Drama of the Commons. Washington, DC: National Academy Press; 2002. p. 41–86.
65. Foss NJ. Leadership, Beliefs and Coordination: An Explorative Discussion. Ind Corp Chang. 2001; 10(2):357–89.
66. Wit A, Wilke H. Subordinates' endorsement of an allocating leader in a commons dilemma: An equity theoretical approach. J Econ Psychol. 1988; 9(2):151–68.

67. Wilke HAM. Greed, Efficiency and Fairness in Resource Management Situations. Eur Rev Soc Psychol. 1991; 2(1):165–87.
68. Armitage D, Marschke M, Plummer R. Adaptive co-management and the paradox of learning. Glob Environ Chang. 2008; 18(1):86–98.
69. Biggs R, Blenckner T, Folke C, Gordon L, Norström A, Nyström M, et al. Regime Shifts. In: Hastings A, Gross L, editors. Encyclopedia of Theoretical Ecology. Berkeley, CA: University of California Press; 2012. p. 609–17.
70. Scheffer M, Carpenter S, Foley JA, Folke C, Walker B. Catastrophic shifts in ecosystems. Nature. 2001; 413(6856):591–6. PMID: 11595939
